# Assessing the impacts of COVID-19 vaccination programme’s timing and speed on health benefits, cost-effectiveness, and relative affordability in 27 African countries

**DOI:** 10.1186/s12916-023-02784-z

**Published:** 2023-03-08

**Authors:** Yang Liu, Simon R. Procter, Carl A. B. Pearson, Andrés Madriz Montero, Sergio Torres-Rueda, Elias Asfaw, Benjamin Uzochukwu, Tom Drake, Eleanor Bergren, Rosalind M. Eggo, Francis Ruiz, Nicaise Ndembi, Justice Nonvignon, Mark Jit, Anna Vassall

**Affiliations:** 1 Department of Infectious Disease Epidemiology, Faculty of Epidemiology and Population Health, London School of Hygiene and Tropical Medicine, Keppel St, London, UK; 2 Centre for Mathematical Modelling of Infectious Diseases, London School of Hygiene and Tropical Medicine, Keppel St, London, UK; 3 South African DSI-NRF Centre of Excellence in Epidemiological Modelling and Analysis, Stellenbosch University, Stellenbosch, Republic of South Africa; 4 Department of Global Health & Development, Faculty of Public Health and Policy, London School of Hygiene and Tropical Medicine, Keppel St, London, UK; 5 Health Economics Programme, Africa Centres for Disease Control and Prevention, Addis Ababa, Ethiopia; 6 Department of Community Medicine, University of Nigeria Nsukka, Enugu Campus, Enugu, Nigeria; 7 Centre for Global Development, Great Peter House, Abbey Gardens, Great College St, London, UK; 8 Institute of Human Virology, University of Maryland School of Medicine, 725 W Lombard St, Baltimore, MD USA; 9 Africa Centres for Disease Control and Prevention, Addis Ababa, Ethiopia; 10 School of Public Health, University of Ghana, Legon, Ghana

**Keywords:** Vaccination, COVID-19 | SARS-CoV-2, Economic evaluation, Affordability, Mathematical models, Decision-making, Programme evaluation, Public health interventions

## Abstract

**Background:**

The COVID-19 vaccine supply shortage in 2021 constrained roll-out efforts in Africa while populations experienced waves of epidemics. As supply improves, a key question is whether vaccination remains an impactful and cost-effective strategy given changes in the timing of implementation.

**Methods:**

We assessed the impact of vaccination programme timing using an epidemiological and economic model. We fitted an age-specific dynamic transmission model to reported COVID-19 deaths in 27 African countries to approximate existing immunity resulting from infection before substantial vaccine roll-out. We then projected health outcomes (from symptomatic cases to overall disability-adjusted life years (DALYs) averted) for different programme start dates (01 January to 01 December 2021, *n* = 12) and roll-out rates (slow, medium, fast; 275, 826, and 2066 doses/million population-day, respectively) for viral vector and mRNA vaccines by the end of 2022. Roll-out rates used were derived from observed uptake trajectories in this region. Vaccination programmes were assumed to prioritise those above 60 years before other adults.

We collected data on vaccine delivery costs, calculated incremental cost-effectiveness ratios (ICERs) compared to no vaccine use, and compared these ICERs to GDP per capita. We additionally calculated a relative affordability measure of vaccination programmes to assess potential nonmarginal budget impacts.

**Results:**

Vaccination programmes with early start dates yielded the most health benefits and lowest ICERs compared to those with late starts. While producing the most health benefits, fast vaccine roll-out did not always result in the lowest ICERs. The highest marginal effectiveness within vaccination programmes was found among older adults. High country income groups, high proportions of populations over 60 years or non-susceptible at the start of vaccination programmes are associated with low ICERs relative to GDP per capita. Most vaccination programmes with small ICERs relative to GDP per capita were also relatively affordable.

**Conclusion:**

Although ICERs increased significantly as vaccination programmes were delayed, programmes starting late in 2021 may still generate low ICERs and manageable affordability measures. Looking forward, lower vaccine purchasing costs and vaccines with improved efficacies can help increase the economic value of COVID-19 vaccination programmes.

**Supplementary Information:**

The online version contains supplementary material available at 10.1186/s12916-023-02784-z.

## Background

Since COVID-19 vaccines were first authorised in late 2020, many countries achieved considerable coverage in a short period [1], reducing disease and economic burdens enormously. However, accruing COVID-19 vaccine coverage in many African countries has been slow before 2022, with one of the largest contributors being vaccine shortage [2]. Given roll-out efforts by the end of 2021, it would be a substantial challenge for the region to achieve the 70% coverage target by mid-2022 as set out by the World Health Organization (WHO) and the Africa Centres for Disease Control and Prevention (Africa CDC) [3–5].

The vaccine shortage has eased significantly since early 2022 [4]. By this time, however, many African Union member states had already experienced several epidemic waves involving multiple variants of concern [1, 6]. A key question that these countries face is whether rolling out COVID-19 vaccines remains an impactful strategy representing good value for money, having missed key windows of opportunities from early- to mid-2021. Continuing vaccination raises issues in respect of both cost-effectiveness and affordability in the region due to potentially low health impacts and high health opportunity costs to other health services due to nonmarginal budgetary impact [7, 8].

This study aims to inform future decisions about vaccine roll-out and investment by retrospectively examining the impacts of implementation timing and speed on COVID-19 transmission using a combined epidemiological and economic modelling approach. This approach allowed us to factor in the potentially high seroprevalence of the region, the emergence of multiple variants of concerns, and population characteristics key to the transmission of SARS-CoV-2 (e.g. population age structure, contacts, non-pharmaceutical interventions) and the economic evaluation of vaccination programmes (gross domestic product per capita (GDP per capita), general public health expenditures).

Given that the COVID-19 vaccine roll-out occurred relatively late and slowly among African countries, we focused on answering two key questions: (i) how much health impacts and value for money could have been achieved had the vaccine roll-out been earlier and faster? (ii) could late COVID-19 vaccine roll-out efforts (in late 2021) still provide considerable health impacts and value for money? To answer these questions, we evaluated different vaccine roll-out scenarios in terms of health impacts, cost-effectiveness, and relative affordability by vaccine types among Africa Union member states.

This is the first multi-country regional analysis of COVID-19 vaccine strategies for Africa that links epidemiological models and economic evaluation with findings that reflect on between-country heterogeneities. The lessons learned could inform regional decision-makers on future vaccine roll-out decisions in Africa, particularly if reformulated vaccines become available in response to future variants. To our knowledge, this is also the first study to empirically examine the impacts of implementation timing while appraising COVID-19 vaccine policies.

## Methods

We estimated the prevalence of infection-induced protection against COVID-19 by fitting a dynamic transmission model to country-level daily reported COVID-19 deaths. This procedure allowed us to capture different epidemic histories experienced by African Union member states. Cumulative health outcomes and disability-adjusted life years (DALYs) averted associated with different vaccine roll-out scenarios were then simulated. Combining health outcomes with data on the costs of vaccine delivery and COVID-19-related health services, we estimated the overall cost-effectiveness and relative affordability from a health sector perspective by the end of 2022. This section provides essential information to understand the methods. Further details are presented in Additional File 1.

All analyses were done in R (4.1.0). All data used are publicly available. Code and intermediate results can be accessed via our GitHub repository archived at Zenodo [9].

### Characterising vaccine roll-out scenarios

**Fig. 1:**
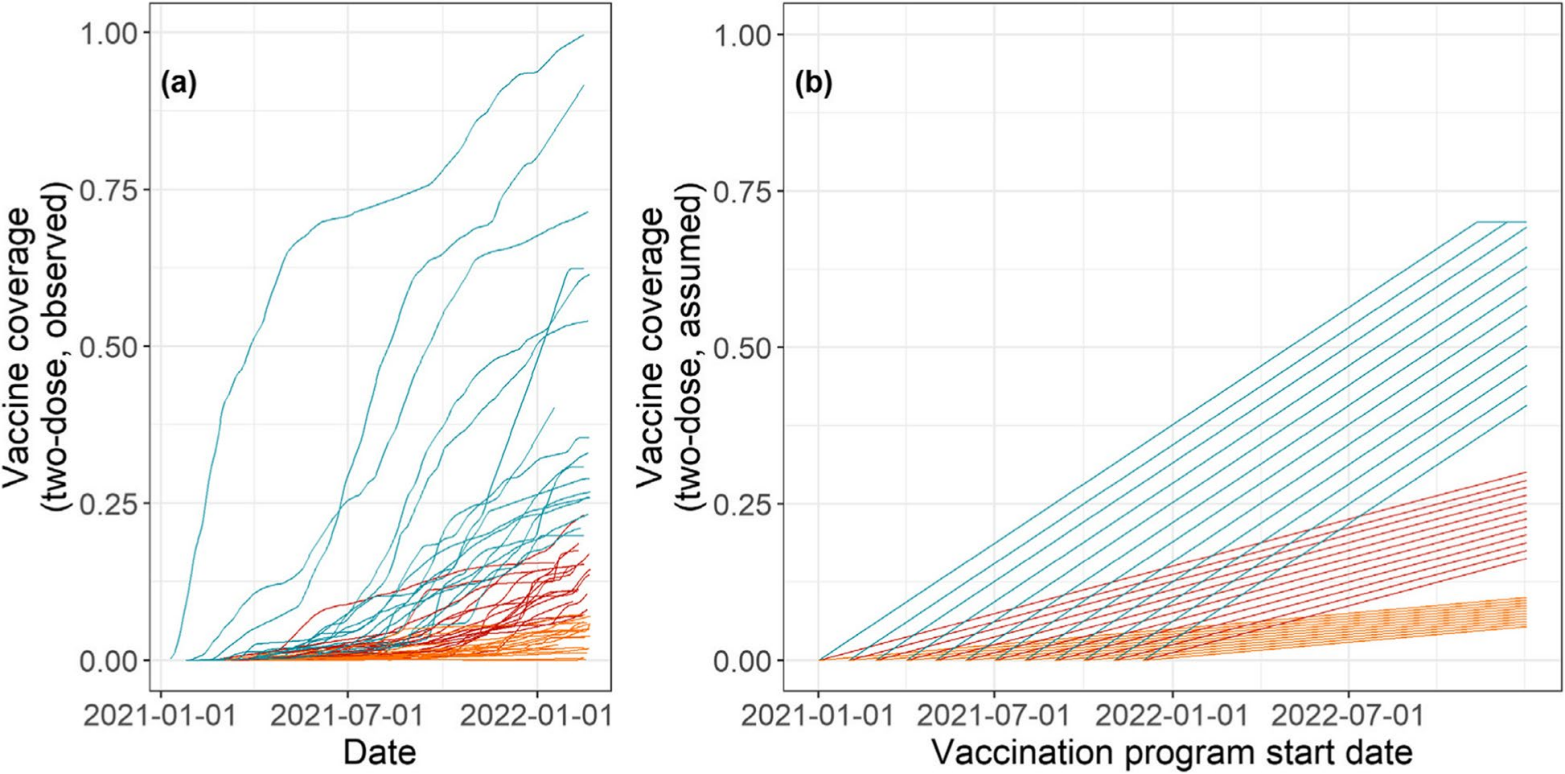
Model parameterisation and vaccine roll-out scenarios setup. (**a**) Observed vaccine uptake among African Union member states. Each line represents a country, and its colour indicates the roll-out rate levels (orange - slow; red - medium; blue - fast). (**b**) Vaccine supply trajectories of 36 vaccine roll-out scenarios (12 programmes starting date × 3 roll-out rates) and the population level vaccine coverage levels they realise

By the time most African Union member states achieved 1% COVID-19 vaccine coverage (August 2021), the United Kingdom (UK) had vaccinated 74.6% (as of 31 August 2021) of its population [1]. By February 2022, vaccine coverage in over 35% of African Union member states was still below 10% (Fig. 1a) [1]. We explored possible alternative vaccine roll-out scenarios that could have taken place in 2021 by varying two parameters: vaccination programme start date and vaccine roll-out rates. The programme start date was defined as the date at which the first person in a country was vaccinated and was varied between 01 January 2021 and 01 December 2021 in 1-month increments. We limited the testing window for start dates to before 2022, as all African Union member states had introduced COVID-19 vaccines by then [1].

Vaccine roll-out rate was defined as the number of doses administered per million population-day. In this study, we varied this parameter along three levels: 275, 826, and 2066 doses/million population-day (hereafter ‘slow’, ‘medium’, and ‘fast’, respectively). These levels were derived from observed vaccine roll-out trajectories using a univariable linear model (cumulative doses per million population-day ~ date) and represent the median vaccine roll-out rates by tertile observed among African Union members [1].

The vaccine roll-out scenarios combining these two parameters (*n* = 12 start dates × 3 roll-out rates) are visualised in Fig. 1b (age-specific example presented in Additional File 1: Fig. S1). We capped the maximum population-level vaccine coverage at 70%, consistent with the target set by the WHO and the Africa CDC [3, 5]. Assuming a two-dose schedule for COVID-19 vaccines, a country with a population size of *n* will receive a maximum of *1.4n* doses of vaccines, likely via early and fast roll-out scenarios.

Older adults (60 + years of age) were prioritised in the vaccine roll-out process based on international guidelines and common practices observed in the region [10, 11]. In the transmission model, older adults were also characterised by higher susceptibility, higher percentages of infection showing symptoms, and high percentages of infections progressing to severe, critical, and fatal outcomes. We assume the maximum vaccine uptake rates among older adults to be 80% and other adults 60% [12].

### Fitting and simulation using a dynamic transmission model

The epidemic model used in this study was an adaptation of CovidM, an age-specific dynamic transmission model of COVID-19, which has been previously used to explore the impact of different vaccination strategies in the UK, European countries, and Pakistan [13–16]. Between-country heterogeneity has been captured by a wide range of health (e.g. age structure, life expectancy, reported COVID-19-related deaths), behaviour (e.g. community mobility, social contacts), economic (e.g. GDP per capita, government public health expenditure, costs), and policy (e.g. COVID-19 response stringency index) factors using local data. The conceptual diagram of the transmission model is presented in Additional File 1: Fig. S2 [14]; the model is further described in Additional file 1: Methods S1 [14, 17–22] and S3 [13, 14, 23–25]; complete parameter tables and their data sources can be found in Additional File 1: Table S1-6 [1, 3, 10–20, 22–44] by category.

We fitted this model to country-level daily reported COVID-19 deaths during 2020–2022, before the wide use of COVID-19 vaccines, using maximum likelihood estimation and differential evolutionary algorithm (full optimisation procedure described in Additional File 1: Methods S2) [14, 45]. The following parameters were estimated: (1) the basic reproduction numbers (R0); (2) infection introduction dates; (3) COVID-19 death reporting rate; and (4) variants of concern introduction dates. The fitting process aims at approximating the immune profiles (i.e. the levels of infection-induced immunity by age and the distribution of non-susceptible individuals within the population by compartments) against SARS-CoV-2 before vaccine roll-out.

Among the 55 African Union member states, we identified 27 with sufficient data for model fitting.^1^ The remaining 28 member states were excluded from further analysis due to either data sparsity (i.e. <  = 10 deaths/day throughout the fitting period, *n* = 26) or potential reporting artefacts (i.e. with single day accounting for > 5% of the cumulative COVID-19 deaths since early 2020, *n* = 2). These countries, despite of interest among regional decision makers, are spatially clustered (Additional File 1: Fig. S3), which makes reasonable extrapolation unfeasible. The epidemic curves describing COVID-19-related deaths among 27 members included in this analysis are presented in Additional File 1: Fig. S4-6 [1].

We explored vaccine roll-out scenarios with either mRNA vaccines (with characteristics similar to those of the Pfizer-BioNTech COVID-19 vaccines) or viral vector vaccines (with characteristics similar to those of Oxford/AstraZeneca vaccines). Six types of vaccine effect mechanisms were incorporated into the model: infection-, disease-, severe case-, critical case-, mortality-reducing, and onward transmission-blocking.

The vaccine efficacy estimates we used are presented in Table 1. An alternative efficacy estimate set based on the lower bounds of their uncertainty ranges in existing literature was tested as a sensitivity analysis. We assumed infection-induced immunity among naïve individuals to last 3 years on average [22]. Given the time horizon of this study, we did not consider the protection waning among fully vaccinated individuals. Decreased vaccine efficacy due to immune evasiveness (e.g. against Omicron) was implemented through changes in susceptibility among vaccinated individuals. However, we assumed immune evasiveness only affects infection- and disease-reducing vaccine efficacies—other types of vaccine efficacies remained constant.

**Table 1:** Vaccine efficacy estimates by vaccine type

Outcome	Vaccine efficacy Base case set | sensitivity analysis set			
	mRNA vaccines		Viral vector vaccines	
	1st dose	2nd dose	1st dose	2nd dose
Infection	0.7 | 0.55	0.85 | 0.7	0.7 | 0.55	0.75 | 0.65
Cases	0.7 | 0.55	0.9 | 0.85	0.7 | 0.55	0.8 | 0.7
Severe cases	0.85 | 0.75	0.95 | 0.9	0.85 | 0.75	0.9 | 0.8
Critical cases	0.85 | 0.73	0.95 | 0.93	0.85 | 0.75	0.93 | 0.78
Deaths	0.85 | 0.7	0.95 | 0.95	0.85 | 0.75	0.95 | 0.75
Onward transmission	0.47 | 0.47	0.47 | 0.47	0.47 | 0.47	0.47 | 0.47

In the context of this study, cases are defined as symptomatic infections; severe cases are defined as those that require hospitalisation; critical cases are defined as those that require critical/intensive care units at some point during their hospital stay. This includes cases that eventually proceed to death as their final outcome. These estimates are broadly in line with Barnard et al. [16]. However, Barnard et al. did not provide estimates for efficacy against critical cases [16]. Based on the relative relationship we observed between vaccine efficacy against severe cases (i.e. hospitalisations of at least 2 days long with acute respiratory infection (ARI) code in the primary diagnosis field), critical cases (i.e. hospitalisations of at least 2 days long with ARI code in primary diagnosis field and with either oxygen, ventilation, or intensive care unit use), and deaths reported in the UK Health Security Agency Vaccine Surveillance Report (week 12) [46], we assumed the vaccine efficacy against critical cases to be the average of that against severe cases and that against death. The alternative estimates used in the sensitivity analysis (i.e. the lower limits from their estimated uncertainty ranges) are from the same report [46]

We projected four health outcomes under each vaccine roll-out scenario: (a) symptomatic infections; (b) severe cases that require hospitalisation; (c) critical cases that require intensive care unit (ICU) admission; and (d) deaths (see Additional File 1: Table S2 for parameter sources and Additional File 1: Methods S4 [14, 15, 23] for technical details). All outcomes were aggregated from 01 January 2021 to 31 December 2022. We kept the time horizon relatively short due to the uncertainty around future emerging variants. However, vaccination programmes that start late may require time to present impacts. We extended the time horizon to 30 June 2023 as a sensitivity analysis.

### Calculating DALYs

We used projected health outcomes to calculate DALYs incurred, which capture both mortality and morbidity burden. Mortality outcomes were converted to years of life lost (YLLs) using country-specific life expectancy [26]. Morbidity outcomes (symptomatic infections, severe cases, critical cases and those who experience long-term health effects (“Long-COVID”)) were converted to years lived with disability (YLDs). We discounted YLLs over remaining life expectancy and additionally discounted DALYs according to the year in which COVID-19 health outcomes occurred. In line with the WHO guidelines on the economic evaluation of immunisation programmes, we used an annual discount rate of 3% [47]. More information on calculating DALYs can be found in Additional File 1: Methods S5-6 [26, 48–51].

### Estimating the vaccine delivery and health service costs

We collected data on country-specific unit costs per vaccine delivered by vaccine type from a health sector perspective in three countries (Ethiopia (a low-income country), Nigeria (a lower-middle-income country), and South Africa (an upper-middle-income country)). For South Africa, we included observed vaccine delivery costs. However, at the time of this study, realistic vaccine delivery costs were not available for any other country in the region. Thus, in this study, we used a normative (i.e. per-protocol) ingredient-based (i.e. itemised) approach (see Additional File 1: Methods S7) [52–54]. The unit cost includes vaccine purchasing costs and related components and activities involved in the planning and delivery of the vaccine (e.g. planning and coordination, cold chain, transportation, and waste disposal) [52]. Costs were then validated by experts knowledgeable of country-level immunisation efforts. In the case of Ethiopia and Nigeria, this validation step was part of the health technology assessment process used to support decision-making around COVID-19 vaccinations.

We found that the unit cost of delivering mRNA vaccines to be substantially higher than that of viral vector vaccines (Table 2). These differences are driven by vaccine purchasing costs (see itemised costs by country, activity, and component in Additional File 1: Methods S7, Additional File 1: Tables S7-8)—for which we used the lowest purchasing price reported in the public domain for each vaccine [55, 56]. We extrapolated these vaccine unit costs for other countries (see Additional File 1: Methods S8 [57–60] for extrapolation methods; this process did not differentiate by country-specific purchasing agreements) and other vaccination programme setups (i.e. programme duration and daily vaccine roll-out rate, see Additional File 1: Methods S9 for the extrapolation methods, and Additional File 1: Table S9 for sample extrapolation results). The extrapolation results indicate that faster vaccine roll-out rates are associated with lower vaccine unit costs due to fixed costs being spread between more vaccine doses (Table 2). Our costing approach assumed no increases in the price of scarce resources (e.g. healthcare workforce).

**Table 2:** Vaccine unit costs by roll-out scenario and vaccine type

Roll-out scenario		Vaccine type	Lower limit ($)	First quartile ($)	Median ($)	Third quartile ($)	Upper limit ($)
Start date	Roll-out rate						
01 August 2021	Medium	Viral vector vaccine	4.96	6.04	6.92	8.44	15.40
	Fast	Viral vector vaccine	2.54	3.61	4.49	6.02	12.97
	Medium	mRNA vaccine	14.2	15.27	16.15	17.68	24.63
	Fast	mRNA vaccine	11.8	12.84	13.72	15.25	22.20

Summary of the vaccine unit costs by country (*n* = 27) estimated for vaccine roll-out efforts starting in 01 August 2021 using medium and fast roll-out rates by vaccine types. The underlying methods have been described in Additional File 1: Methods S7-9. Raw data behind these estimates were presented in Additional File 1: Table S7-8. Further sample estimates by country are presented in Additional File 1: Table S9. In this study, vaccine delivery unit costs differ by country, vaccine type, vaccine roll-out rates, and programme duration

For COVID-19-related health service costs, we used previously published country-specific estimates (Table 3) and lengths of hospital stay [31, 48]. All costs were converted to US$2020 using GDP deflators [61]. Similarly to DALYs, costs were discounted by 3% in line with WHO guidelines [47].

**Table 3:** Health service costs

Health service endpoints	Lower limit ($)	First quartile ($)	Median ($)	Third quartile ($)	Upper limit ($)
Home-based care	4.84	17.22	22.11	44.52	266.24
Hospital-based care for severe cases	27.02	33.65	37.08	45.74	170.99
Hospital-based care for critical cases	156.63	245.10	277.57	336.06	1685.98
Management of fatal cases	65.29	65.29	65.29	65.29	65.29

Summary of the vaccine unit costs by country (*n* = 27) estimated for vaccine roll-out efforts starting 01 August 2021 using medium and fast roll-out rates by vaccine types. The underlying methods have been described in Additional File 1: Methods S7-9. Further sample estimates by country are presented in Additional File 1: Table S9

### Measuring cost-effectiveness and relative affordability

We estimated incremental cost-effectiveness ratios (ICERs) from a healthcare payer perspective for different vaccination scenarios compared to a no-vaccination scenario by dividing the differences in total costs between alternatives (including vaccine delivery and COVID-19 health service costs) by the differences in DALYs incurred. Vaccination costs may be paid by country governments or external donors. To allow for between-country comparison, we calculated ICER as a proportion of GDP per capita and thus intrinsically assuming a proportion of GDP per capita may approximate the underlying health opportunity costs [62, 63].

We did not fix a single cost-effectiveness threshold and instead focused our discussion on ICER as a proportion of GDP per capita. This is because there are no generally accepted cost-effectiveness thresholds for any country in Africa or, indeed, most of the world [64]. Furthermore, COVID-19 vaccination is likely to have a large budget impact, and hence the thresholds calculated at the margin of budgets may not always apply [7, 8, 65]. In practice, a cost-effectiveness analysis using an overestimated cost-effectiveness threshold may incur high health opportunity costs in budget-constrained settings as the programme under evaluation could affect existing public health activities. In low- and middle-income countries, substantial uncertain remains around the appropriate cost-effectiveness threshold [63, 66]. In this study, for simplicity, we assumed conditions where ICERs account for more than 50% of GDP per capita are less likely to be considered cost-effective [63, 66].

We ran additional multivariable regression analyses to explore the country-specific metrics that can link to ICERs as proportions of GDP per capita. We tested population size [26], proportion of population over 60 years [26], proportion of population non-susceptible at the start of vaccine roll-out (model fitted in this study), vaccine unit cost (estimated in this study), government general health expenditure [33], and income group [34].

In this study, we defined the relative affordability of a vaccine programme in a given year as the annual incremental cost of the programme divided by annual government general health expenditure [7, 8]. The annual incremental cost of a programme is calculated by the sum of vaccination programme costs and the corresponding COVID-19-related healthcare costs minus the COVID-19-related healthcare costs without any vaccine during the entire time horizon and then standardised to the annual level. Since the external donor contribution may be uncertain (or redirected through different funding schemes) in the COVID-19 era, only domestic healthcare budgets were considered.

We estimated ICER as a proportion of GDP per capita and relative affordability to evaluate COVID-19 vaccination programmes. The two underlying streams of data used, GDP per capita and government general health expenditure, are not strictly correlated. As a result, cost-effectiveness and affordability outcomes may lead to different conclusions—vaccination programmes may be cost-effective but not affordable, and vice versa.

## Results

**Fig. 2:**
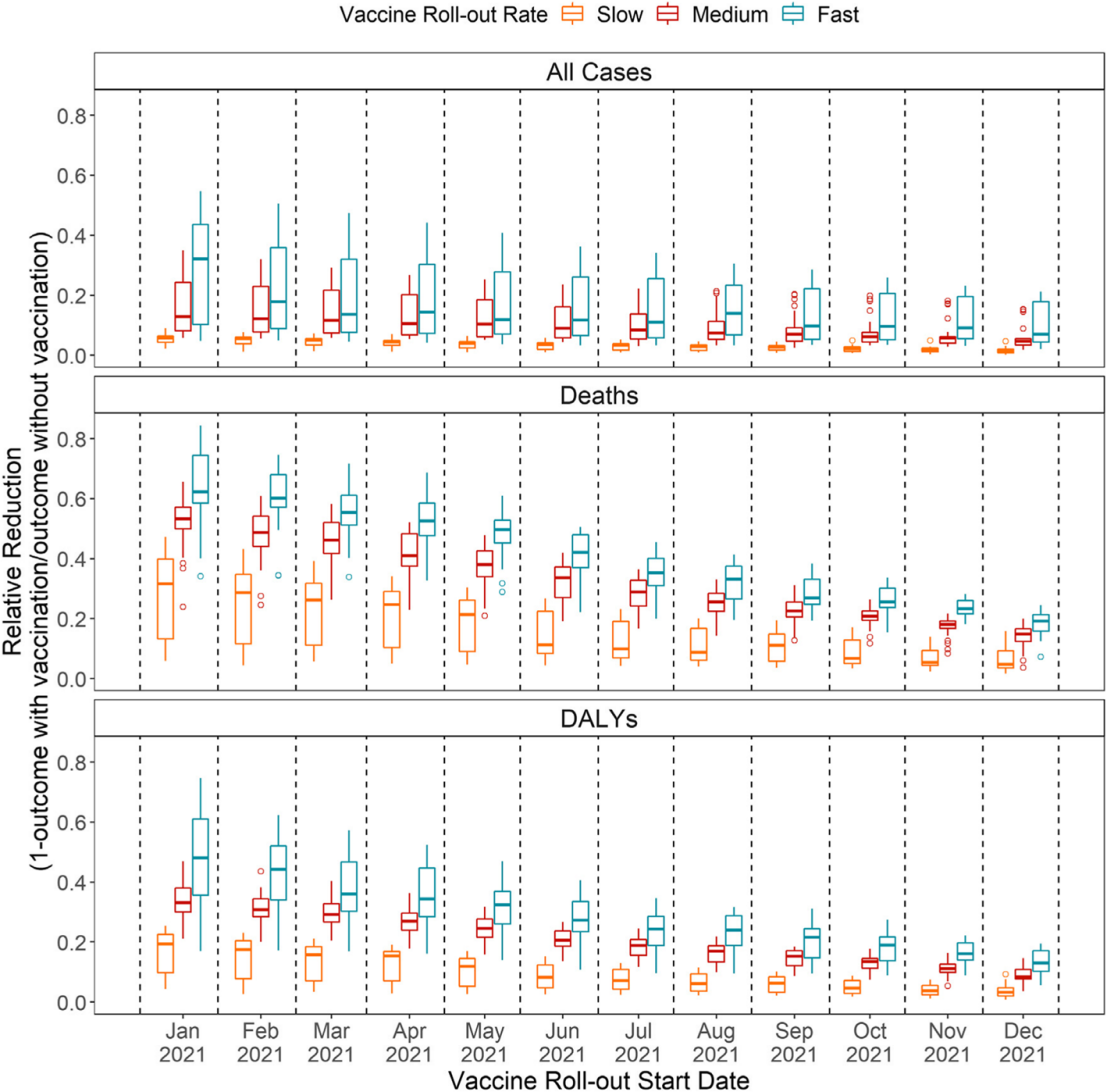
Outcomes associated with different vaccine roll-out scenarios for 27 African Union Members using viral vector vaccines. This figure presents the relative reduction in outcomes as a result of different vaccine roll-out scenarios (i.e. combinations of vaccination programme start dates and vaccine roll-out rates). Relative reduction is defined as (1 - outcome with vaccination/outcome without vaccination). Greater relative reductions indicate more effective vaccine roll-out scenarios. Results for intermediate health outcomes and for using mRNA vaccines can be found in Additional File 1: Fig. S8-10. DALYs: disability-adjusted life years

The fitted model could broadly reproduce the observed epidemic history of COVID-19 before vaccine roll-out by country (see Additional File 1: Fig. S4-6 for model fits and S7 for fitted parameter distributions). The projected impacts of viral vector vaccines on cases, deaths, and DALYs by roll-out scenario are presented in Fig. 2 (see Additional File 1: Fig. S8-10 for intermediate results and results for using mRNA vaccines). We calculated relative reduction in disease burden instead of absolute differences to allow the comparison across countries with varying population sizes.

For a given vaccination programme start date, faster roll-out rates are associated with a substantially greater disease burden averted compared to the no-vaccination scenario. For example, compared to the no-vaccination scenario, vaccination programmes starting in August 2021 (when more than half of African Union member states with data (*n* = 53) reached 1% population-level vaccine coverage) could have reduced deaths by an average of 11.06% [median = 8.80%, interquartile range: 5.87–16.20%; *n* = 27 (countries)] under slow roll-out, 24.19% [24.43%, 21.78–27.18%] under medium roll-out, and 31.29% [32.57%, 25.72–36.68%] under fast roll-out.

For a given vaccine roll-out rate, earlier roll-out start dates are associated with greater disease burden averted compared to the no-vaccination scenario. For example, under medium roll-out, starting in January could have reduced deaths by a mean of 50.30% [51.29%, 48.97–54.95%]. However, starting the same programme in August 2021 could only have reduced deaths by a mean of 24.19% [24.32%, 21.78–27.18%]. These decreasing trends in relative impact reduction are steeper for more severe outcomes, which reflects different vaccine efficacies by outcome and the features of emerging variants of concern.

**Fig. 3:**
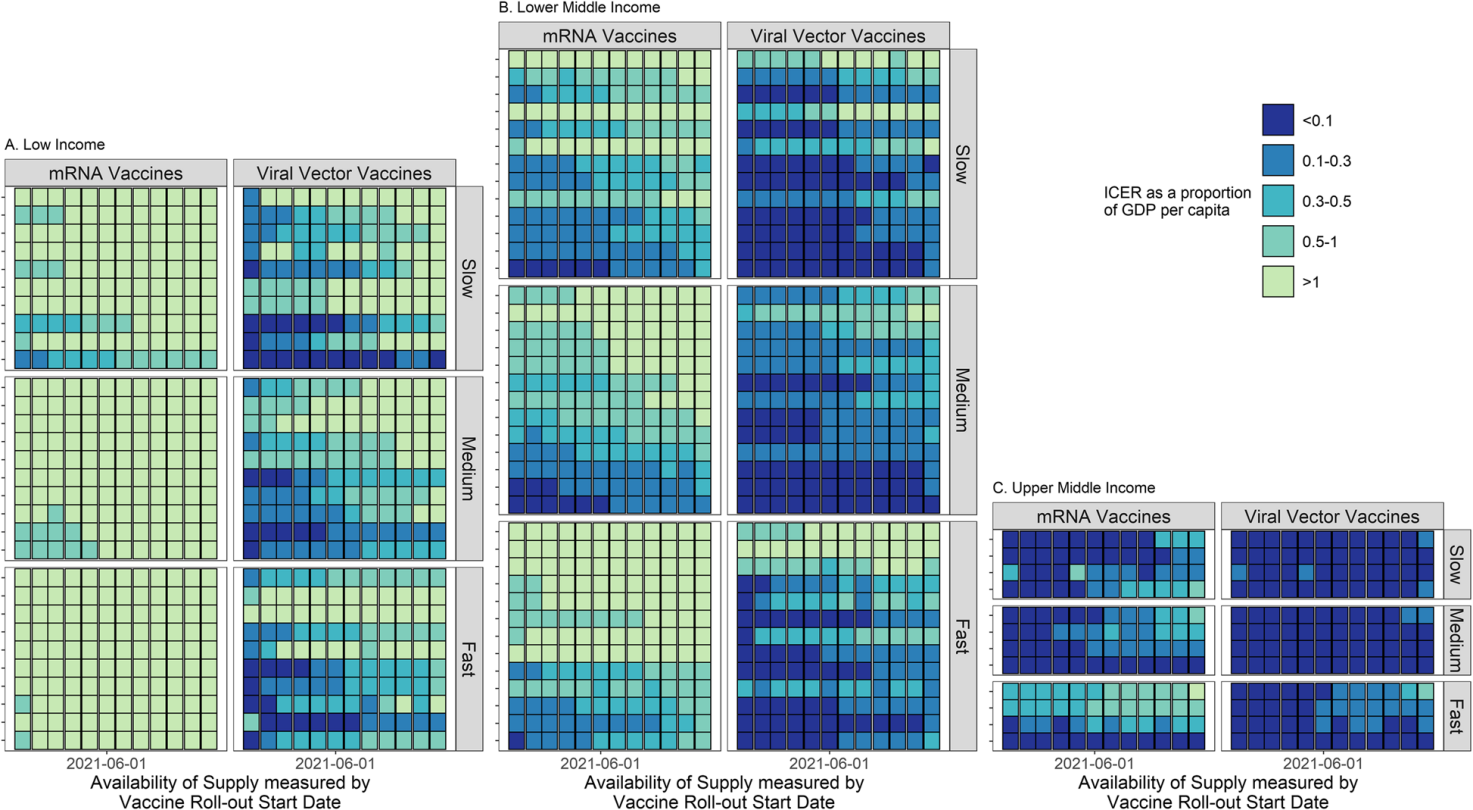
ICERs in relation to GDP per capita by vaccine roll-out scenarios. **A.** Low-income countries. **B.** Lower-middle-income countries. **C.** Upper-middle-income countries. Each *y*-axis tick represents a country; the *y*-axis is arranged based on ICERs as proportions of GDP per capita. The baseline for comparison is the no-vaccination scenario (01 January 2021–31 December 2022)

Given the same start date, faster programmes may be associated with lower ICERs in relation to GDP per capita (Fig. 3). At all income levels, medium and fast roll-out scenarios are associated with lower ICERs relative to GDP per capita compared to slow. However, medium roll-out scenarios may result in lower ICERs relative to GDP per capita compared to fast. This can be explained by differential marginal effectiveness by age group as the fast roll-out scenarios quickly move into younger adult populations (which are less cost-effective to vaccinate) after vaccinating older adults (Additional File 1: Methods S10). Compared to older adults, younger adults are less susceptible and less likely to progress to more severe outcomes.

The results of multivariable regression analysis linking country characteristics and ICER as a proportion of GDP per capita revealed that a country’s income group was statistically significant (using type I error rate of 0.05) and had the largest explanatory power (i.e. effect size, Additional File 1: Fig. S11). Higher income groups were associated with low ICERs as proportions of GDP per capita. In upper-middle-income countries, ICERs account for, on average, − 2.52% and 20% of GDP per capita using viral vector and mRNA vaccines, respectively (Fig. 3C). Vaccination programmes may be cost-saving. In low-income countries, ICERs account for, on average, 82.59% and 313.52% of GDP per capita using viral vector and mRNA vaccines, respectively (Fig. 3A). These vaccination programmes are unlikely to be considered cost-effective. In low-income countries, only viral vector vectors and only when vaccination programmes start in the first half of 2021 could the average ICERs as proportions of GDP per capita have fallen below 50% (Fig. 3A).

The proportion of population above 60 years of age and the proportion of non-susceptible individuals at the start of vaccination programmes were also statistically significant and had the next largest effect sizes. Higher proportions of population above 60 years of age were associated with lower ICERs as proportions of GDP per capita. Higher proportions of non-susceptible population at the start of the vaccination programme were associated with higher ICERs as proportions of GDP per capita. All other variables, except for government general health expenditure, were statistically significantly associated with ICERs as proportions of GDP per capita but had smaller effect sizes relative to the aforementioned three variables.

**Fig. 4:**
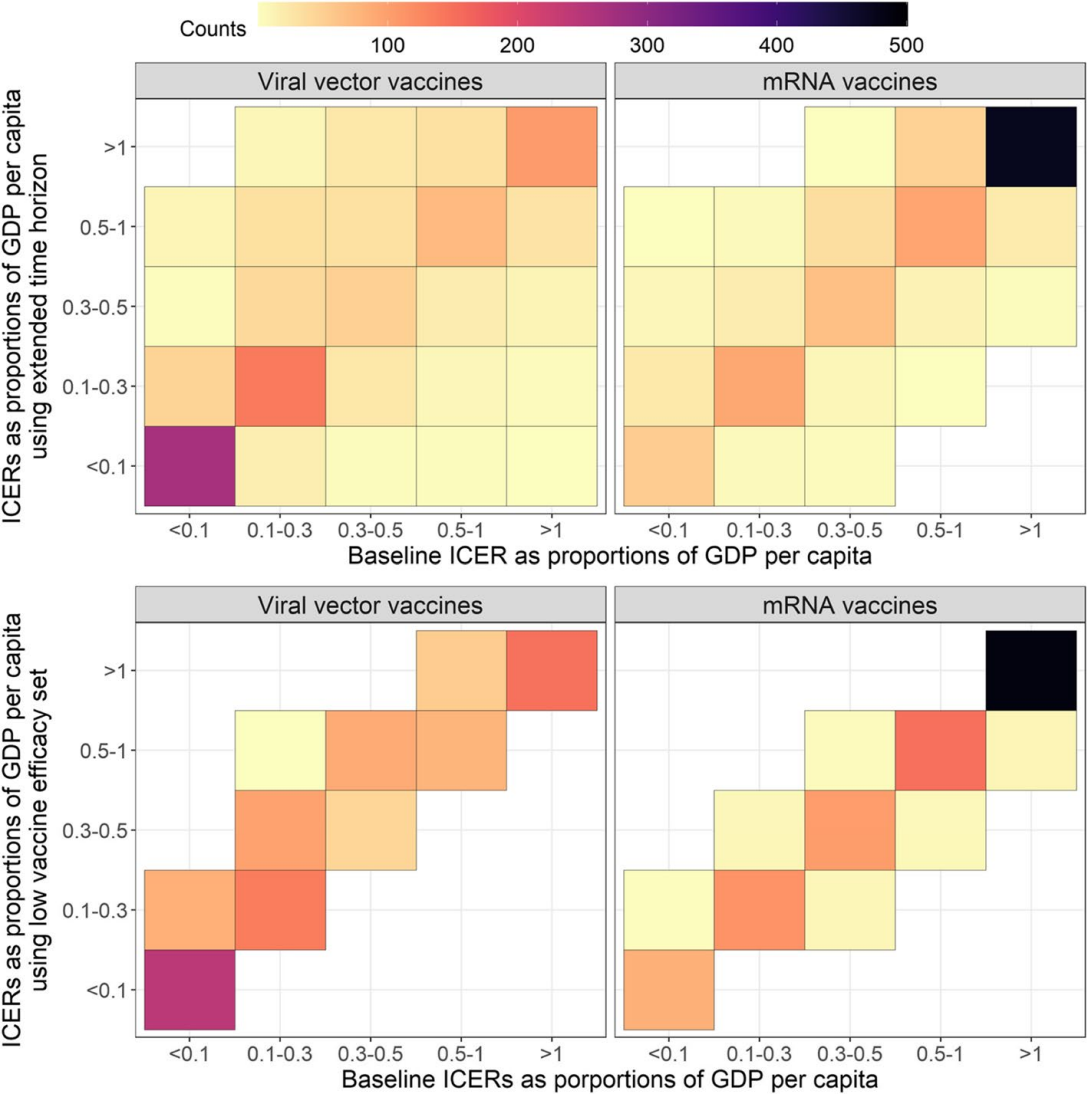
Results from sensitivity analyses. Univariable sensitivity analysis dimensions: (top) time horizon; (bottom) vaccine efficacies. In each panel, a total of 972 data points (27 countries × 36 vaccine roll-out scenarios) have been allocated into each cell based on value, with colours indicating the overall count of data points in that cell. In the bottom right panel, for example, the dark cell on the top right indicates there are approximately 500 country-and-vaccine-roll-out-scenario combinations where the ICER relative to GDP per capita is greater than one in both the baseline analysis and when low vaccine efficacies were implemented. Deviations from the diagonal line indicate there are country-and-vaccine-roll-out-scenario combinations returning different results in terms of ICERs as proportions of GDP per capita resulting from the univariable sensitivity analysis

Our sensitivity analyses around the time horizon show that drawing the line on 31 December 2022 affects how ICERs compare to GDP per capita (Fig. 4, top panels). Of 972 country and roll-out scenario combinations (27 countries × 36 scenarios), 34.88% and 19.65% returned different results when we extended the time horizon by 6 months while using viral vector and mRNA vaccines, respectively. These differences are expressed as deviations from the diagonal line in Fig. 4. Deviations above the diagonal line indicate by extending the time horizon ICERs got larger. Stratified analyses revealed that most deviations above the diagonal lines had late start dates; most deviations below had early start dates. An extended time horizon makes the observed contrast in ICER over start date (Fig. 3) more extreme, indicating the robustness of our results to the analytical window.

Using lower vaccine efficacies affects ICERs as proportions of GDP per capita to a much lesser extent. We expect low vaccine efficacy to be associated with high medical costs and low overall QALYs averted compared to the no vaccine used scenario and thus high ICERs as proportions to GDP per capita. This is the case with both viral vector and mRNA vaccines (Fig. 4, bottom panels; non-empty cells exist to the top left of the diagonal line). While using mRNA vaccines, lower vaccine efficacies may lead to lower ICERs as proportions to GDP per capita (Fig. 4, bottom right panel; non-empty cells exist on the bottom right of the diagonal line). In these cases, lower vaccine efficacy leads to larger outbreaks earlier on, resulting in more individuals being protected by hybrid immunity by the time that the more transmissible strain emerges.

The median relative affordability estimates for vaccination programmes involving mRNA vaccines implemented with slow, medium, and fast roll-out rates are 3.85% [interquartile range: 1.26–10.24%], 12.42% [3.43–29.84%], and 26.13% [7.82–61.61%], respectively, and for viral vector vaccines 1.09% [0.18–3.02%], 3.24% [0.61–6.61%], and 5.28% [0.70% -10.98%], respectively.

**Fig. 5:**
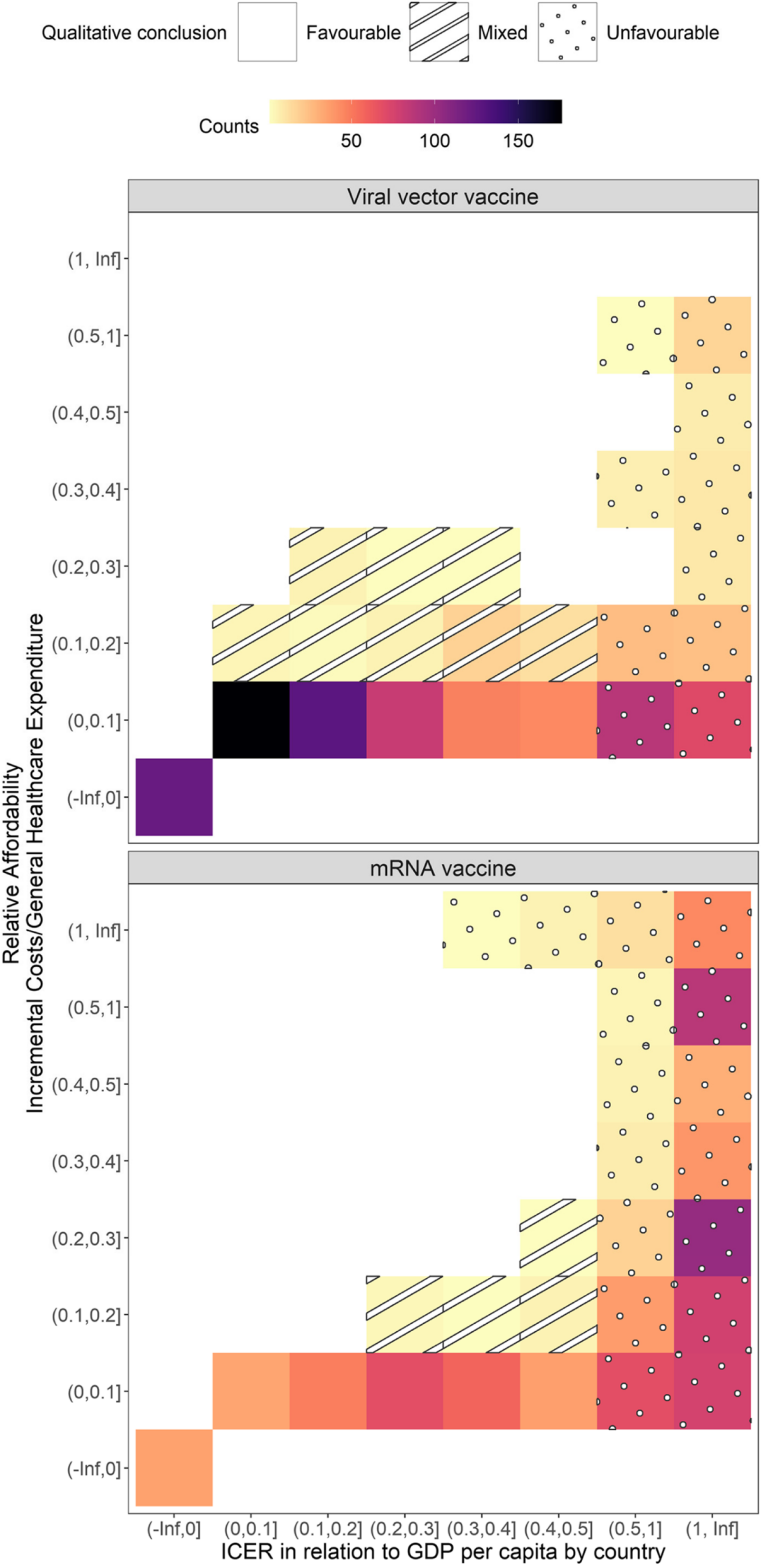
ICERs as proportions of GDP per capita and relative affordability of COVID-19 vaccine roll-out scenarios. In each panel, a total of 900 data points (25 countries × 36 vaccine roll-out scenarios) have been allocated in each cell, with colours indicating the overall count of data points in that cell. Only 25 of 27 countries with ICER results also had relative affordability results. Data on government general health expenditure for Somalia and Libya were not available. In the top panel, for example, there are over 150 country-and-vaccine-roll-out-scenario combinations where the ICERs account for 0–10% of the GDP per capita and where incremental costs of the vaccination programmes account for 0–10% of the country’s general healthcare expenditure. An unfavourable conclusion (dotted pattern cells) is extremely unaffordable (> 100% of general healthcare expenditure) or one where ICERs as proportions of GDP per capita are too high (> 0.5); a favourable conclusion (pattern-free cells) is one where ICERs as proportions of GDP per capita are low (< = 0.5) and programme costs are also low (< = 10% of general healthcare expenditure); a mixed conclusion (striped pattern cells) is one where ICERs as proportions of GDP per capita are low (< = 0.5), but programme costs are high (> = 10% of general healthcare expenditure)

In the context of low- and middle-income countries, scenarios where ICERs account for more than 50% of GDP per capita are less likely to be cost-effective (Fig. 5, dotted cells) [63, 66]. Scenarios where ICERs account for less than 50% of GDP per capita and where programme cost less than 10% of government general health expenditure may be favourable policy options (Fig. 5, pattern-free cells). Some scenarios have negative net costs and ICERs, indicating they are cost-saving and health-improving. Other scenarios (5.22% and 1.11% of country-and-roll-out-scenario combinations for viral vector and mRNA vaccines, respectively) have low ICERs as proportions of GDP per capita but cost more than 10% of government health expenditure (Fig. 5, striped cells). In these cases, some COVID-19 vaccination programmes may still be cost-effective yet unaffordable. Decision makers may need to adjust their perceived health opportunity costs for a DALY, given COVID-19 is now a new and leading source of health burden [7, 8]. The need for this adjustment may be mitigated by shifting costs to external donors (e.g. via Gavi COVID-19 Vaccines Global Access (or COVAX), Advance Market Commitment) among health sector payers.

Raw estimates behind the results presented above (including incremental costs, incremental DALYs, ICER, GDP per capita, and ICERs as proportions of GDP per capita) by each scenario and country can be downloaded from our GitHub repository.

## Discussion

Using a combined epidemiological and economic modelling approach, we explored how the timing and speed of implementation (i.e. vaccination programme start date and vaccine roll-out rates) affect vaccination programmes’ health benefits, cost-effectiveness, and relative affordability among African countries. We found that vaccination programmes with earlier start dates lead to greater health benefits and are more likely to result in small ICERs in relation to GDP per capita. Fast vaccination roll-out leads to the greatest health benefits but does not always lead to the smallest ICERs in relation to GDP per capita. This is due to differential marginal effectiveness by age. Fast scenarios quickly move beyond the vulnerable and thus prioritised population (defined as 60 + years of age in this study, see Additional File 1: Methods S10). These results should not be extrapolated to other parts of the world without further validation; these results also should not be extrapolated to other vaccine products due to the fundamental differences in the underlying epidemiology.

Higher income groups and greater proportions of population over 60 years of age were associated with lower ICERs as proportions to GDP per capita. Greater proportions of populations non-susceptible at the start of vaccination programmes were associated with higher ICERs as proportions of GDP per capita. The proportions of populations non-susceptible at the start of vaccination programmes were estimated by fitting a dynamic transmission model in this study. In practice, decision-making based on this metric requires accurate serological surveys, which is particularly challenging to conduct in pandemic response settings.

There were missed opportunities in early- to mid-2021 for COVID-19 vaccination to save more lives in Africa. However, even with a late start, certain vaccine types and roll-out rates combinations may still generate relatively low ICERs. Two factors could further increase the economic value of vaccination programmes. First, our results are based on the known immune dynamics of SARS-CoV-2 and the performance of current COVID-19 vaccines. New vaccines with better effectiveness against emerging variants could decrease ICER by increasing health benefits. Emerging variants with the protection advantage of vaccine-induced immunity over infection-induced immunity may have the same effect.

Second, our results show that vaccine roll-out scenarios are much more likely to be cost-effective when using viral vector vaccines than mRNA vaccines. The relatively similar vaccine efficacy estimates against severe health outcomes (Table 1) and the drastically different vaccine unit costs (Table 2) may explain this. The main driver of vaccine unit costs is vaccine price. Some﻿ low-income countries may struggle to find COVID-19 vaccination programmes cost-effective after factoring in budget constraints and potential health opportunity costs. This highlights the importance of achieving even lower vaccine purchasing prices than those we assumed in this analysis. In this study, we estimated that the price of viral vector vaccines may be reduced by 10.32–28.69% and mRNA vaccines by 36.91-60.96% to reduce ICERs to 10–50% of GDP per capita (Additional File 1: Fig. S12).

We found a small number of countries where ICERs were small in relation to GDP per capita, yet the programmes were relatively unaffordable. The implication is that the investment in managing COVID-19, a new and leading cause of disease burden, may risk opportunity costs for existing public health and health service issues. Our affordability estimates are from a health sector perspective and have not explicitly accounted for the potential externalities on the local health systems and essential health services. Unless development partners provide subsidies that compensate for these effects, the perceived opportunity cost of a DALY, which is often approximated using a fraction of GDP per capita, may need to be further adjusted down from what we already know them for in low- and middle-income settings (which are low) [7, 8, 63, 66]. Affordability and ICERs in relation to GDP per capita are two of many metrics that stakeholders base their decisions on while planning for vaccine programmes.

This is the first study to examine the role of timing in the cost-effectiveness of COVID-19 vaccination programmes. We highlighted key issues to consider in the next pandemic (or next epidemic waves triggered by emerging variants of concern) to come. Timing has already been shown to influence the health impacts of vaccination programmes. Incorporating it into economic evaluation would lead to more accurate and realistic cost-effectiveness assessments [67, 68]. We fit a mathematical model with a range of plausible vaccine effect mechanisms to the COVID-19 epidemic histories in 27 African Union member states. Most existing modelling studies on the economic evaluation of COVID-19 vaccination strategies have either focused on a single country [69] or city [15] or used hypothetical epidemic histories that are not based on data from any country [70], which hinders the interpretability and generalisability of their results.

It is not the objective of this study to directly inform COVID-19 vaccine policies in individual countries. A regional analysis constrained us to use only data streams available in all countries under consideration. Thus, although we provided country-specific estimates in our GitHub repository, we caution national-level stakeholders against interpreting these results out of the regional context. Evidence for informing policies in individual countries may be generated by better-fitted transmission models (e.g. channelling more locally available data streams, such as more detailed non-pharmaceutical interventions and ICU occupancy) using the modelling framework we presented here (code available through our GitHub repository).

Our study has several limitations. First, our epidemic model has not captured the full complexity of the immune dynamics against SARS-CoV-2, which involves both vaccination and infections. For example, our model assumed that vaccinated individuals who had prior infection histories were completely protected against SARS-CoV-2. This design is intended to ensure that most individuals within the model receive a maximum of two doses of vaccine. However, we cannot capture a small number of breakthrough infections that may still happen. These tend to be mild cases and, thus, should not alter our results substantially [71]. Despite best efforts, there may be additional factors that may affect the epidemiology of COVID-19 that we have not implemented in this model (e.g. baseline prevalence of chronic health conditions). Second, we assumed the vaccine supply shortage was the only reason for slow uptake. In practice, uptake may also be slowed down by factors such as vaccine hesitancy [72]. The vaccine delivery unit costs may have been underestimated in this study as the vaccination programmes may require greater efforts in terms of social mobilisation. Third, we did not use observed costs of different vaccination programmes, as this data is not yet available. The normative approach (i.e. per-protocol) we took did not account for the resource wastes incurred in operations or the increased prices of scarce resources that may be associated with the pace of the roll-out, healthcare utilisation rate, or potential structural changes to local health systems as a result of COVID-19 pandemic response. As a result, ICERs may have been biased. Fourth, the cost-effectiveness analysis we presented is from a country’s perspective and does not consider COVID-19 transmission in nearby countries (e.g. transmission spillover). Finally, our analysis took on a health sector perspective and did not account for societal elements such as changes in productivity or the preservation of key functions (e.g. labour market, emergence response, healthcare, and education). These societal elements specific to the COVID-19 pandemic have been difficult to estimate and may vary substantially between countries—additional data and research on these elements could improve the comprehensiveness of vaccine policy evaluation in the future.

## Conclusions

We assessed the impact of COVID-19 vaccination programmes’ timing and speed on the health benefits, cost-effectiveness, and relative affordability in 27 African countries. We found that earlier vaccination programmes led to greater health benefits and generated lower ICERs in relation to GDP per capita. Although fast vaccination programmes yielded the greatest health benefits, they did not always generate the lowest ICERs in relation to GDP per capita as they covered larger proportions of individuals who were less vulnerable. Lower vaccine purchasing costs and improved vaccine efficacies may improve the overall cost-effectiveness and affordability of COVID-19 vaccination programmes.

## Supplementary Information

**Additional file 1: Supplemental Figures: Figure S1.** Vaccine roll-out trajectories by age group. **Figure S2.** Diagram of Transmission Model Structure. **Figure S3.** Countries included and excluded from this analysis. **Figure S4-6.** Performance of model fitting process, Part 1-3 (in alphabetical order). **Figure S7.** Fitted parameters. **Figure S8-10.** Health Outcomes Associated with Vaccine Roll-out Scenarios (by vaccine type and by health outcome). **Figure S11.** The association between the proportion of DALYs averted attributable to older adults and the performance of medium and fast scenarios. **Figure S11.** Effect sizes estimated in the multivariable regression model linking country characteristics to ICERs as proportions of GDP per capita. **Figure S12.** Target vaccine price under different perceived cost-effectiveness thresholds. **Supplemental Tables: Table S1.** Model Equations. **Table S2.** Epidemic and Healthcare Process Parameters. **Table S3.** Additional Data Sources. **Table S4.** Other vaccine and vaccination programme characteristics. **Table S5.** Variants of Concern Introduction. **Table S6.** List of countries with fitted models. **Table S7.** Itemised cost per dose per activity for base countries - viral vector / AstraZeneca-like vaccine. **Table S8.** Itemised Cost per dose per activity for base countries - mRNA / Pfizer-like vaccine. **Table S9.** Cost per dose for countries with fitted models. **Table S10.** CHEERS 2022 Checklist. **Supplemental Methods:** **Methods S1.** Further Model Descriptions. **Methods S2.** Fitting Process. **Methods S3.** Characterising behavioural change using data on non-pharmaceutical intervention and mobility. **Methods S4.** Calculating COVID-19 severe, critical, and death cases. **Methods S5.** Lengths of Stay (LoSs). **Methods S6.** Calculating Disability-adjusted Life Years (DALYs). **Methods S7.** Estimating Vaccine Delivery Costs. **Methods S8.** Extrapolating unit costs from base countries to other countries in Africa. **Methods S9.** Extrapolating vaccine unit costs for different roll-out scenarios. **Methods S10.** ICERs and Proportions of DALYs averted by those above 60 years.

## Data Availability

We used publicly available aggregate data and provided appropriate citations. The CovidM modelling framework has been published previously and is available at https://github.com/nicholasdavies/covidm-alpha. All code used and country-specific intermediate results, original a GitHub repository, have been archived at Zenodo as Liu et al. (9). A CHEERS checklist (2022 version) is presented in Additional File 1: Table S10. CHEERS: Consolidated Health Economic Evaluation Reporting Standards.

## Abbreviations

Africa CDC: Africa Centres for Disease Control and Prevention
ARI: Acute respiratory infection
CHEERS: Consolidated Health Economic Evaluation Reporting Standards
DALY: Disability-adjusted life year
GDP: Gross domestic product
ICER: Incremental cost-effectiveness ratio
ICU: Intensive care unit
UK: United Kingdom
WHO: World Health Organization
YLDs: Years lived with disability
YLLs: Years of life lost

## Footnotes

1 In alphabetical order: People’s Democratic Republic of Algeria, Republic of Angola, Republic of Botswana, Republic of Cameroon, Democratic Republic of the Congo, Arab Republic of Egypt, Eswatini, Federal Democratic Republic of Ethiopia, Republic of Ghana, Republic of Kenya, Republic of Liberia, Libya, Republic of Madagascar, Republic of Malawi, Kingdom of Morocco, Republic of Mozambique, Republic of Namibia, Federal Republic of Nigeria, Republic of Rwanda, Republic of Senegal, Somali Republic, Republic of South Africa, Republic of The Sudan, Tunisian Republic, Republic of Uganda, Republic of Zambia, Republic of Zimbabwe.
