# Supplementary material for: Assessing the impacts of COVID-19 vaccination programme’s timing and speed on health benefits, cost-effectiveness, and relative affordability in 27 African countries
Source: BMC Med. 2023 Mar 8;21:85. doi: 10.1186/s12916-023-02784-z (PMC9991879; doi:10.1186/s12916-023-02784-z)
Supplement: Supplementary file 1 — Additional file 1: Supplemental Figures: Figure S1. Vaccine roll-out trajectories by age group. Figure S2. Diagram of Transmission Model Structure. Figure S3. Countries included and excluded from this analysis. Figure S4-6. Performance of model fitting process, Part 1-3 (in alphabetical order). Figure S7. Fitted parameters. Figure S8-10. Health Outcomes Associated with Vaccine Roll-out Scenarios (by vaccine type and by health outcome). Figure S11. The association between the proportion of DALYs averted attributable to older adults and the performance of medium and fast scenarios. Figure S11. Effect sizes estimated in the multivariable regression model linking country characteristics to ICERs as proportions of GDP per capita. Figure S12. Target vaccine price under different perceived cost-effectiveness thresholds. Supplemental Tables: Table S1. Model Equations. Table S2. Epidemic and Healthcare Process Parameters. Table S3. Additional Data Sources. Table S4. Other vaccine and vaccination programme characteristics. Table S5. Variants of Concern Introduction. Table S6. List of countries with fitted models. Table S7. Itemised cost per dose per activity for base countries - viral vector / AstraZeneca-like vaccine. Table S8. Itemised Cost per dose per activity for base countries - mRNA / Pfizer-like vaccine. Table S9. Cost per dose for countries with fitted models. Table S10. CHEERS 2022 Checklist. Supplemental Methods: Methods S1. Further Model Descriptions. Methods S2. Fitting Process. Methods S3. Characterising behavioural change using data on non-pharmaceutical intervention and mobility. Methods S4. Calculating COVID-19 severe, critical, and death cases. Methods S5. Lengths of Stay (LoSs). Methods S6. Calculating Disability-adjusted Life Years (DALYs). Methods S7. Estimating Vaccine Delivery Costs. Methods S8. Extrapolating unit costs from base countries to other countries in Africa. Methods S9. Extrapolating vaccine unit costs for different roll-out scenarios. Method [file 12916_2023_2784_MOESM1_ESM.docx]

[Additional File 1]

**Assessing the impacts of COVID-19 vaccination programme’s timing and speed on health benefits, cost-effectiveness and relative affordability in 27 African Countries**

Yang Liu PhD^1,2, *^, Simon R Procter DPhil^1,2^, Carl AB Pearson PhD^1,2,3^, Andrés Madriz Montero MSc^4^, Sergio Torres-Rueda MSc^4^, Elias Asfaw PhD^5^, Benjamin Uzochukwu MD^6^, Tom Drake DPhil^7^, Eleanor Bergren MSc^4^, Rosalind M Eggo PhD^1,2^, Francis Ruiz MSc^4^, Nicaise Ndembi PhD^8, 9^, Justice Nonvignon PhD^5, 10, #^, Mark Jit PhD^1,2, #^, Anna Vassall PhD^4, #^

1. Department of Infectious Disease Epidemiology, Faculty of Epidemiology and Population Health, London School of Hygiene and Tropical Medicine, Keppel St, London WC1E 7HT, United Kingdom

2. Centre for Mathematical Modelling of Infectious Diseases, London School of Hygiene and Tropical Medicine, Keppel St, London WC1E 7HT, United Kingdom

3. South African DSI-NRF Centre of Excellence in Epidemiological Modelling and Analysis, Stellenbosch University, Stellenbosch, Republic of South Africa

4. Department of Global Health & Development, Faculty of Public Health and Policy, London School of Hygiene and Tropical Medicine, Keppel St, London WC1E 7HT, United Kingdom

5. Health Economics Programme, Africa Centres for Disease Control and Prevention, Addis Ababa, Ethiopia

6. Department of Community Medicine, University of Nigeria Nsukka, Enugu campus

7. Centre for Global Development, Great Peter House, Abbey Gardens, Great College St, London, UK

8. Institute of Human Virology, University of Maryland School of Medicine, 725 W Lombard St, Baltimore, Maryland 21201, United States

9. Africa Centres for Disease Control and Prevention, Addis Ababa, Ethiopia

10. School of Public Health, University of Ghana, Legon, Ghana

* Corresponding author: yang.liu@lshtm.ac.uk

# These authors contributed equally

Keywords: vaccination; COVID-19 | SARS-CoV-2; economic evaluation; affordability; mathematical models; decision-making; programme evaluation; public health interventions

**Table of Content**

[Supplemental Figures 4](#_Toc126686522)

[Figure S1. Vaccine roll-out trajectories by age group 4](#_Toc126686523)

[Figure S2. Diagram of Transmission Model Structure 5](#_Toc126686524)

[Figure S3. Countries included and excluded from this analysis 6](#_Toc126686525)

[Figure S4. Performance of model fitting process, Part 1 (in alphabetical order) 7](#_Toc126686526)

[Figure S5. Performance of model fitting process, Part 2 (in alphabetical order) 8](#_Toc126686527)

[Figure S6. Performance of model fitting process, Part 3 (in alphabetical order) 9](#_Toc126686528)

[Figure S7. Fitted parameters 10](#_Toc126686529)

[Figure S8. Health Outcomes Associated with Different Vaccine Roll-out Scenarios for 27 African Union Members (viral vector vaccines, severe and critical cases) 11](#_Toc126686530)

[Figure S9. Health Outcomes Associated with Different Vaccine Roll-out Scenarios for 27 African Union Members (mRNA vaccines, cases and deaths) 12](#_Toc126686531)

[Figure S10. Health Outcomes Associated with Different Vaccine Roll-out Scenarios for 27 African Union Members (mRNA vaccines, severe and critical cases) 13](#_Toc126686532)

[Figure S11. The association between the proportion of DALYs averted attributable to older adults and the performance of medium and fast scenarios 14](#_Toc126686533)

[Figure S11. Effect sizes estimated in the multi-variable regression model linking country characteristics to ICERs as proportions of GDP per capita 16](#_Toc126686534)

[Figure S12. Target vaccine price under different perceived cost-effectiveness thresholds 17](#_Toc126686535)

[Supplemental Tables 18](#_Toc126686536)

[Table S1. Model Equations 18](#_Toc126686537)

[Table S2. Epidemic and Healthcare Process Parameters* 21](#_Toc126686538)

[Table S3. Additional Data Sources* 23](#_Toc126686539)

[Table S4. Other vaccine and vaccination program characteristics 24](#_Toc126686540)

[Table S5. Variants of Concern Introduction 25](#_Toc126686541)

[Table S6. List of countries with fitted models 26](#_Toc126686542)

[Table S7. Itemized cost per dose per activity for base countries - viral vector / AZ-like vaccine - USD$ 2021 28](#_Toc126686543)

[Table S8. Itemized Cost per dose per activity for base countries - mRNA / Pfizer-like vaccine - USD$ 2021 29](#_Toc126686544)

[Table S9. Cost per dose for countries with fitted models - USD$ 2021 30](#_Toc126686545)

[Table S10. CHEERS 2022 Checklist 32](#_Toc126686546)

[Supplemental Methods 36](#_Toc126686547)

[Methods S1. Further Model Descriptions 36](#_Toc126686548)

[Methods S2. Fitting Process 37](#_Toc126686549)

[Methods S3. Characterising behavioural change using data on non-pharmaceutical intervention and mobility 38](#_Toc126686550)

[Methods S4. Calculating COVID-19 severe, critical, and death cases 39](#_Toc126686551)

[Methods S5. Lengths of Stay (LoSs) 41](#_Toc126686552)

[Methods S6. Calculating Disability-adjusted Life Years (DALYs) 43](#_Toc126686553)

[Methods S7. Estimating Vaccine Delivery Costs 45](#_Toc126686554)

[Methods S8. Extrapolating unit costs from base countries to other countries in Africa 54](#_Toc126686555)

[Methods S9. Extrapolating vaccine unit costs for different roll-out scenarios 56](#_Toc126686556)

[Methods S10. ICERs and Proportions of DALYs averted by those above 60 years 57](#_Toc126686557)

##

## Supplemental Figures

### Figure S1. Vaccine roll-out trajectories by age group


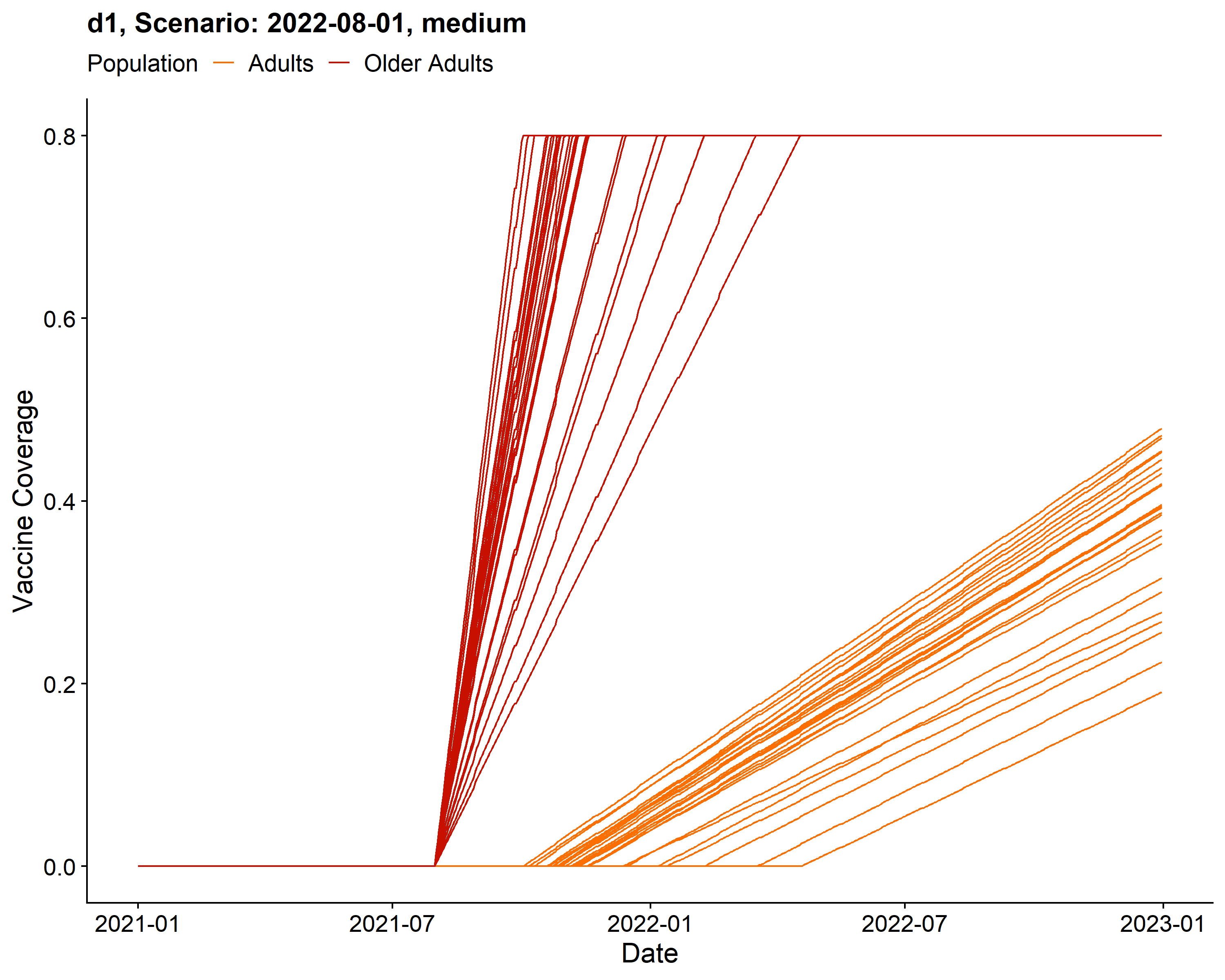


**Caption:** Vaccine roll-out scenarios (n = 36) were combined with dose number (first or second), five-year age bands (0-4 to 75+), and country to generate vaccine roll-out trajectories. In the context of this study, we generated 16 age groups x 2 doses x 36 vaccine roll-out scenarios x 27 countries = 31104 roll-out trajectories in the base case. Due to differences in population age structure, the phase transition between vaccinating older adults and vaccinating adults occurred at different times in each country. In this figure, we demonstrate this concept using the first dose, broad age groups (adults and older adults), and one vaccine roll-out scenario (starting on 2021-08-01 using medium rates). Older adults: 60+ years; adults: 20-59 years.

### Figure S2. Diagram of Transmission Model Structure


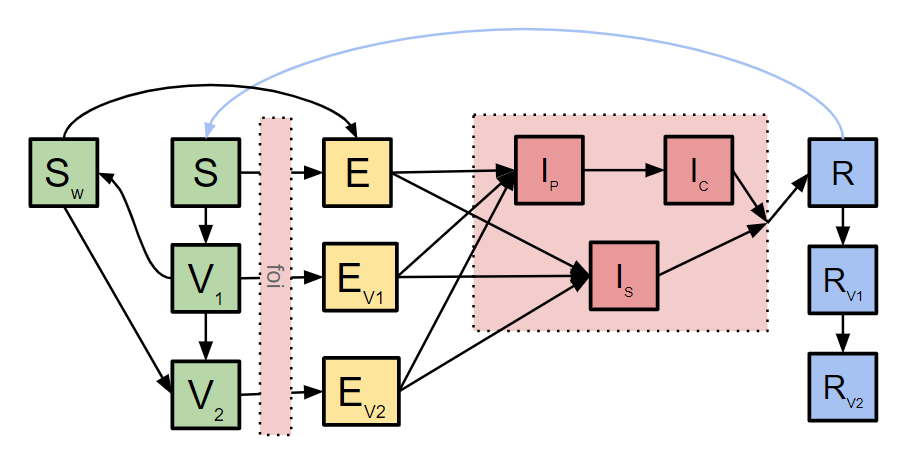


**Caption:** S - Susceptible; V1 - individuals who have received and are protected by one dose of vaccine; Sw - individuals who have received but are not protected by one dose of vaccine; V2 - individuals who have received and are protected by two doses of vaccines; E - exposed individuals; Ev1 - exposed individuals who have previously received and are protected by one dose of vaccines; Ev2 - exposed individuals who have previously received and are protected by two doses of vaccines; Ip - pre-clinical individuals, i.e. individuals who would eventually present symptoms; Ic - clinical and infectious individuals; Is - subclinical (i.e. asymptomatic) and infection individuals; R - removed individuals; Rv1 - individuals with prior infection history and one dose vaccination; Rv2 - individuals with prior infection history and two-dose vaccinations. This model structure and figure have been previously used in Liu et al. [(1)](https://sciwheel.com/work/citation?ids=12801904&pre=&suf=&sa=0)

###

### Figure S3. Countries included and excluded from this analysis

**
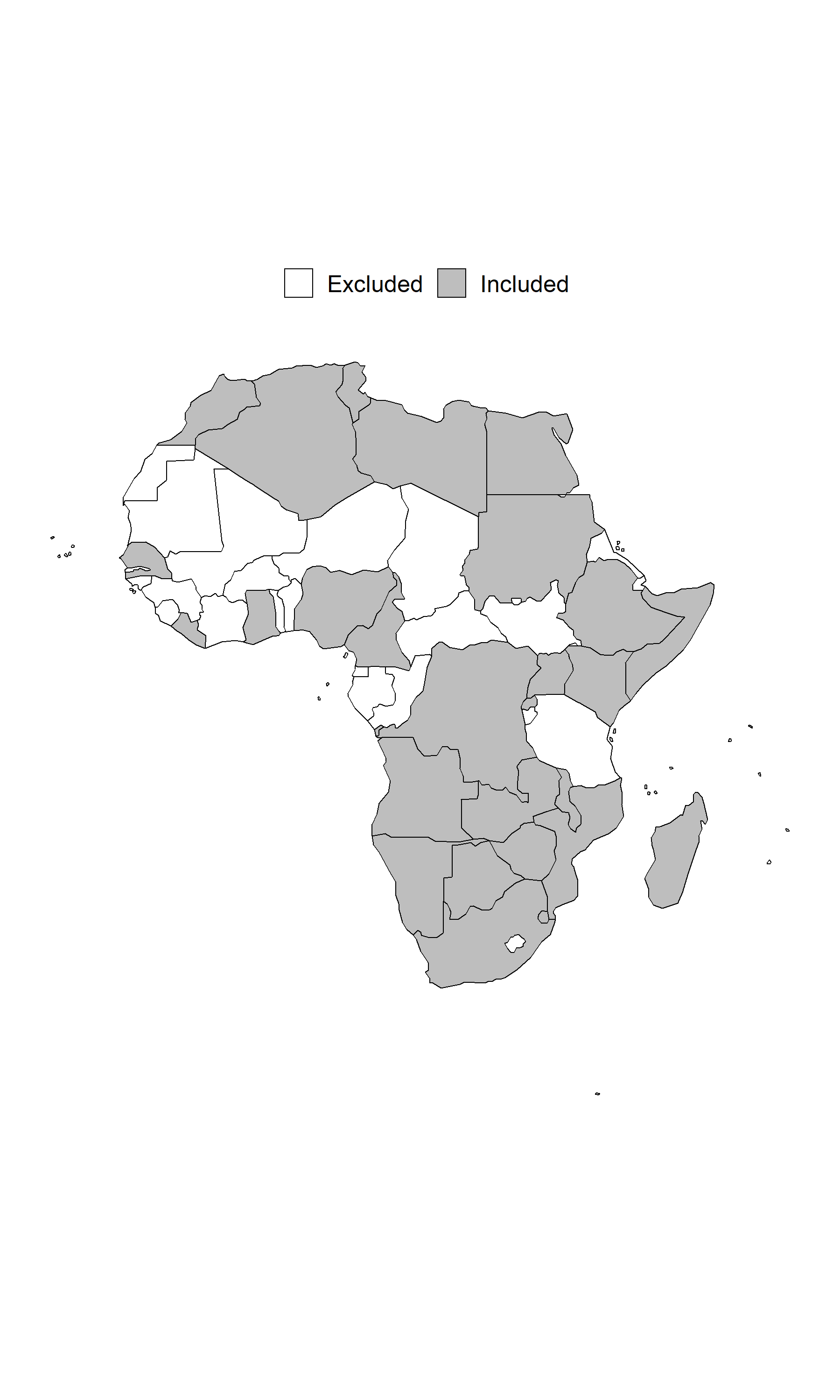
Caption:**

Countries included in the analysis were: Algeria, Angola, Botswana, Cameroon, Dem. Republic of the Congo, Egypt, Eswatini, Ethiopia, Ghana, Kenya, Liberia, Libya, Madagascar, Malawi, Morocco, Mozambique, Namibia, Nigeria, Rwanda, Senegal, Somalia, South Africa, Sudan, Tunisia, Uganda, Zambia, Zimbabwe.

Countries not included were: Republic of Benin, Burkina Faso, Republic of Burundi, Republic of Cabo Verde, Central African Republic, The Republic of Chad, Union of the Comoros, Republic of the Congo, Republic of Cote d'Ivoire, Republic of Djibouti, Republic of Equatorial Guinea, State of Eritrea, Gabonese Republic, Republic of the Gambia, Republic of Guinea, Republic of Guinea-Bissau, Kingdom of Lesotho, Republic of Mali, Republic of Mauritania, Republic of Mauritius, Republic of Niger, Saharawi Arab Democratic Republic, Democratic Republic of São Tomé and Principe, Republic of Seychelles, Republic of Sierra Leone, Republic of South Sudan, United Republic of Tanzania, Togolese Republic.

###
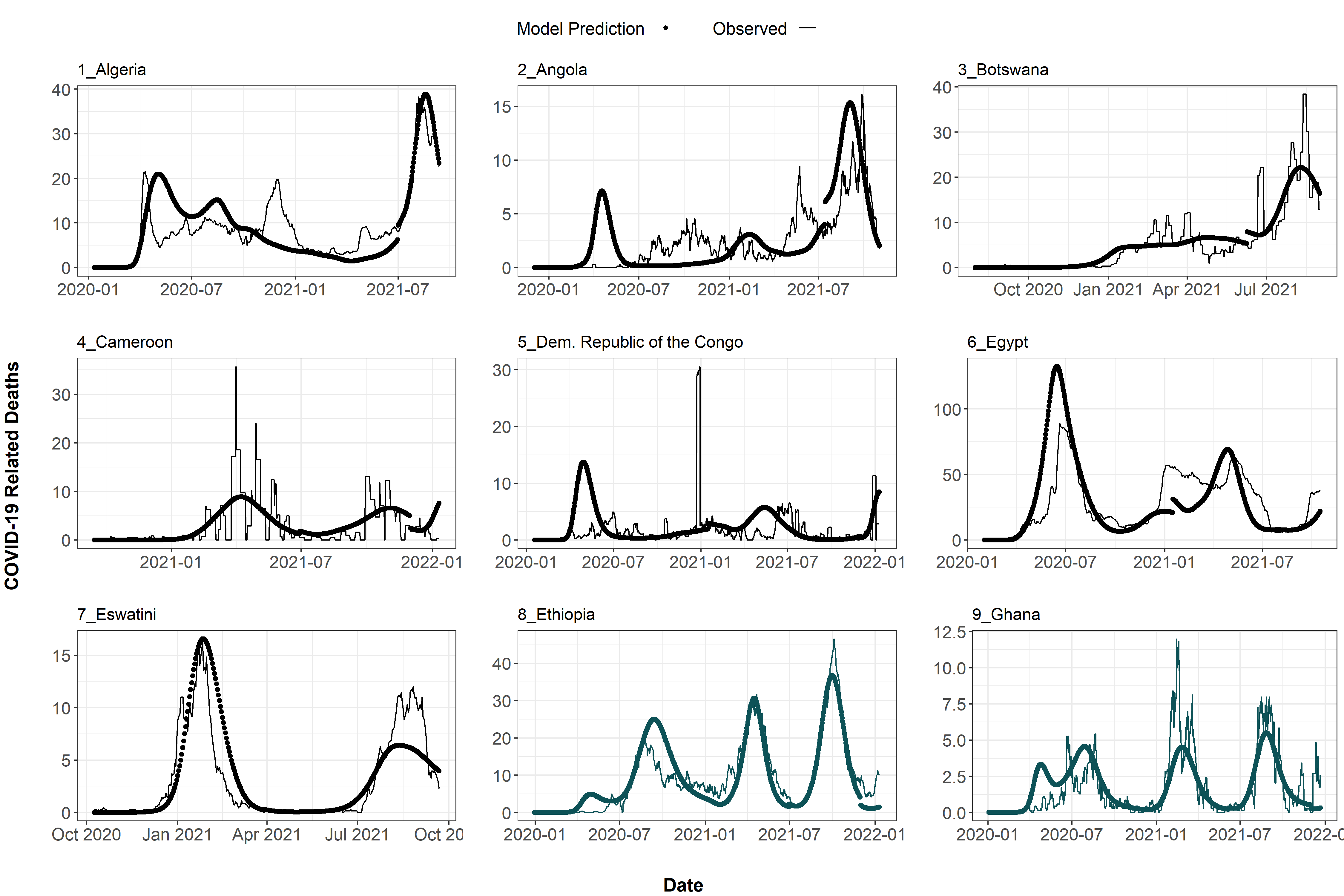
Figure S4. Performance of model fitting process, Part 1 (in alphabetical order)

**Caption:** Points represent predicted values using the fitted model; lines represent observed values accessed through Ritchie et al.[(2)](https://sciwheel.com/work/citation?ids=11703906&pre=&suf=&sa=0&dbf=0) Countries in dark green indicate countries where we fitted for two variants of concern introduction dates. All other countries only have one fitted variants of concern introduction date. The length of the fitting window varies by country and can be found in Additional File 1: Table S6. The methods involved in generating these results are presented in Additional File 1: Methods S1-4.

###
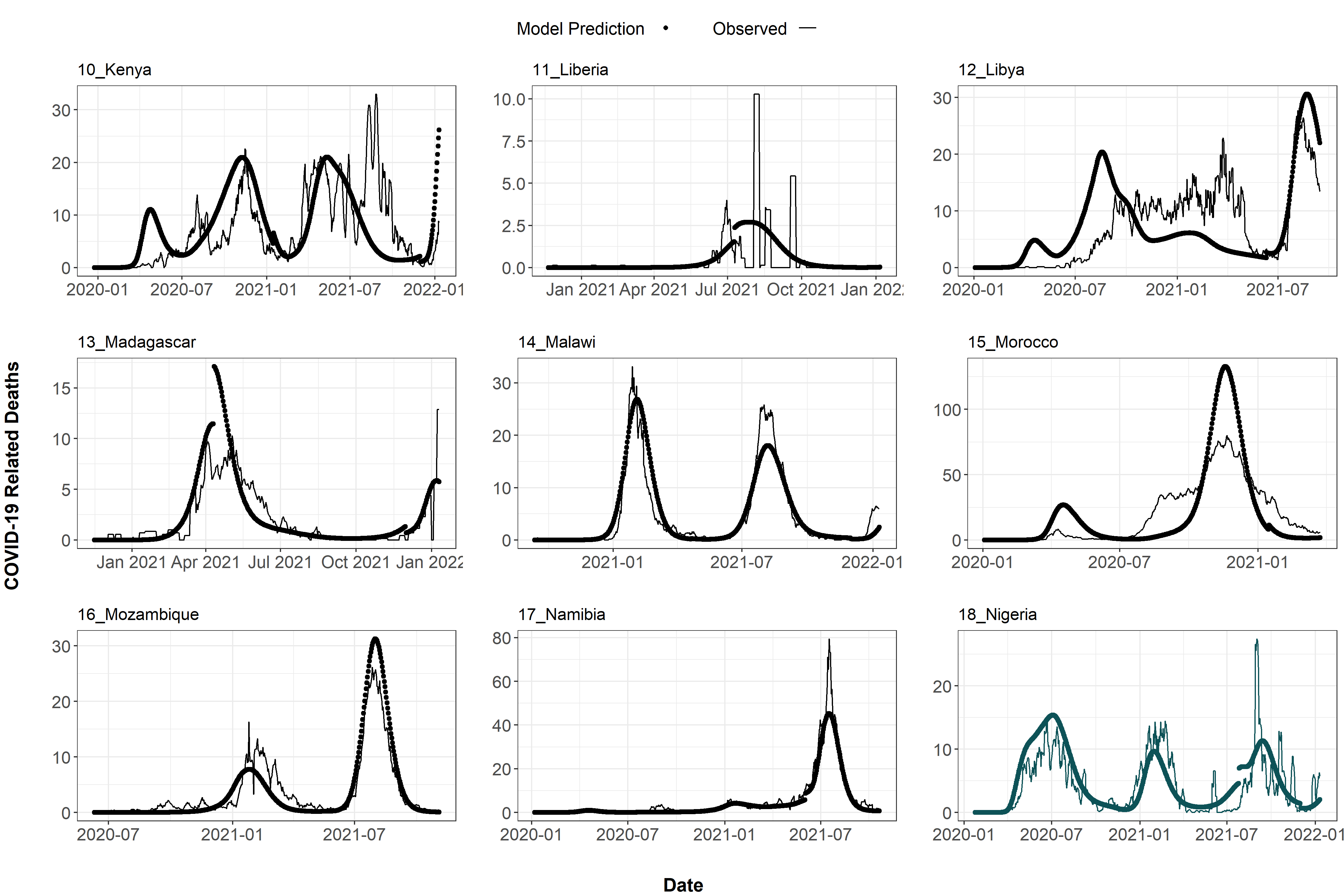
Figure S5. Performance of model fitting process, Part 2 (in alphabetical order)

**Caption:** Points represent predicted values using the fitted model; lines represent observed values accessed through Ritchie et al.[(2)](https://sciwheel.com/work/citation?ids=11703906&pre=&suf=&sa=0&dbf=0) Countries in dark green indicate countries where we fitted for two variants of concern introduction dates. All other countries only have one fitted variants of concern introduction date. The length of the fitting window varies by country and can be found in Additional File 1: Table S5. The methods involved in generating these results are presented in Additional File 1: Methods S1-4.

###
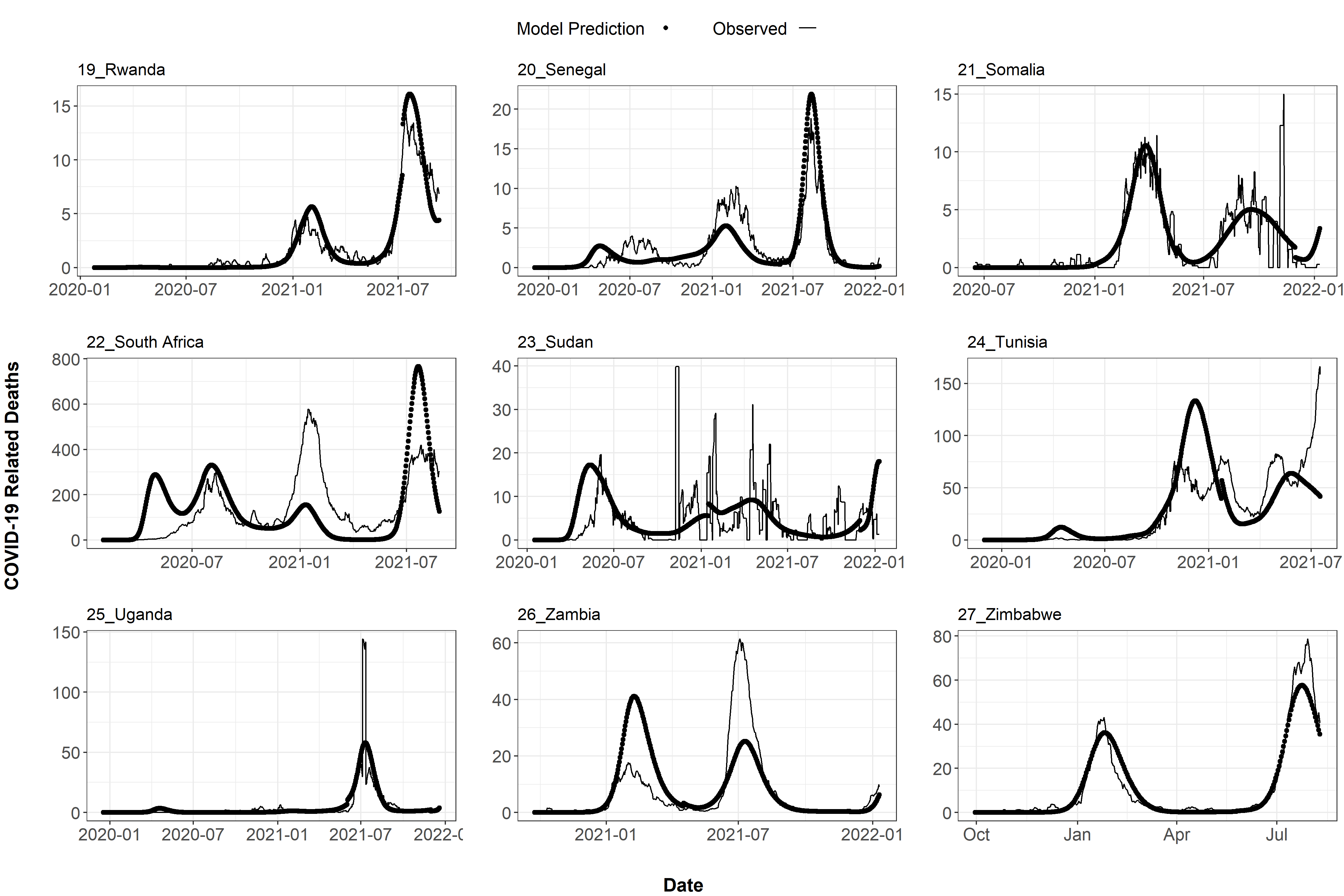
Figure S6. Performance of model fitting process, Part 3 (in alphabetical order)

**Caption:** Points represent predicted values using the fitted model; lines represent observed values accessed through Ritchie et al.[(2)](https://sciwheel.com/work/citation?ids=11703906&pre=&suf=&sa=0&dbf=0) Countries in dark green indicate countries where we fitted for two variants of concern introduction dates. All other countries only have one fitted variants of concern introduction date. The length of the fitting window varies by country and can be found in Additional File 1: Table S5. The methods involved in generating these results are presented in Additional File 1: Methods S1-4.

### Figure S7. Fitted parameters


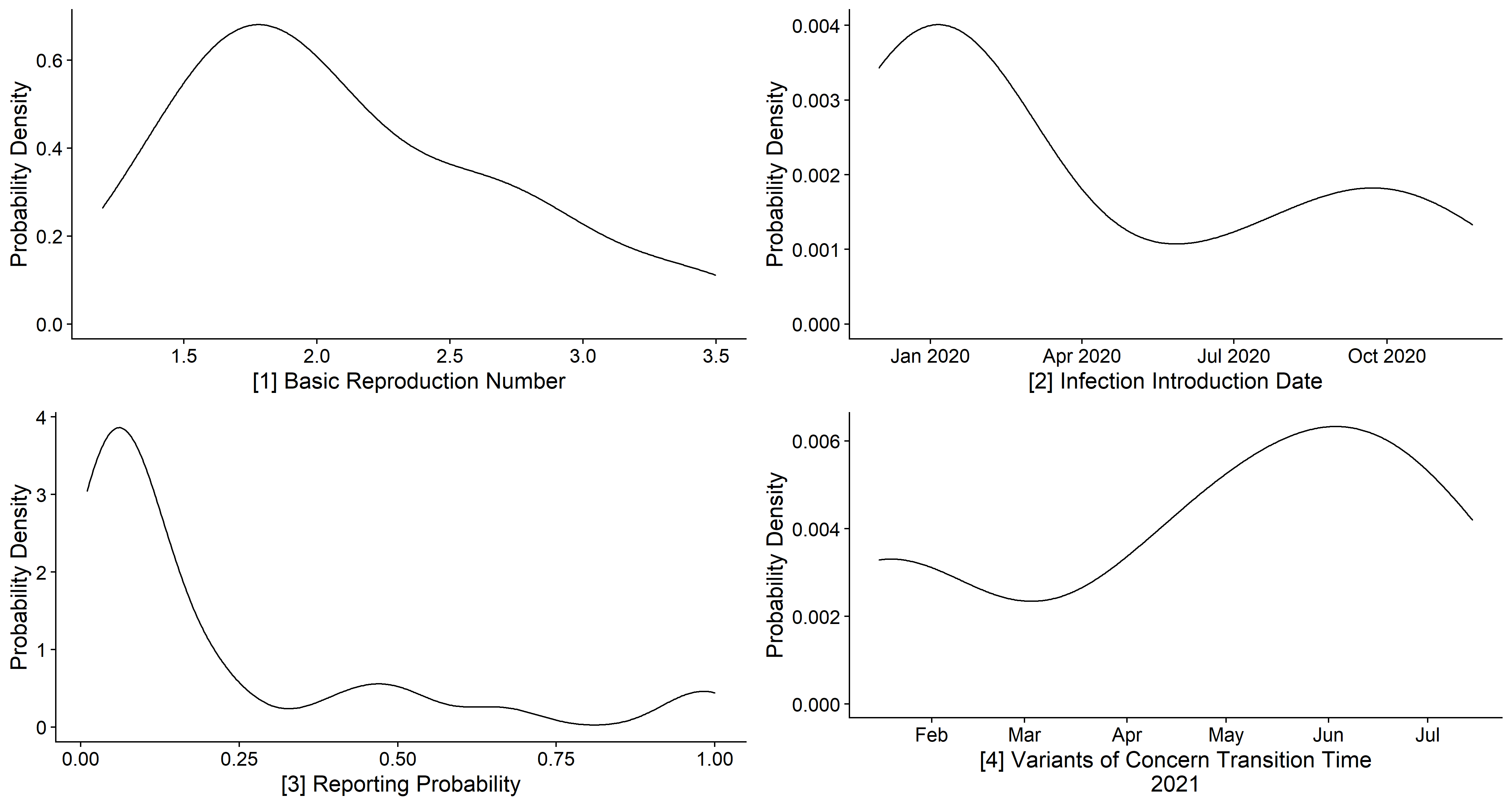
**Caption:** Fitted parameters behind Figure S4-6. Country specific values can be found in our GitHub repository.

### Figure S8. Health Outcomes Associated with Different Vaccine Roll-out Scenarios for 27 African Union Members (viral vector vaccines, severe and critical cases)


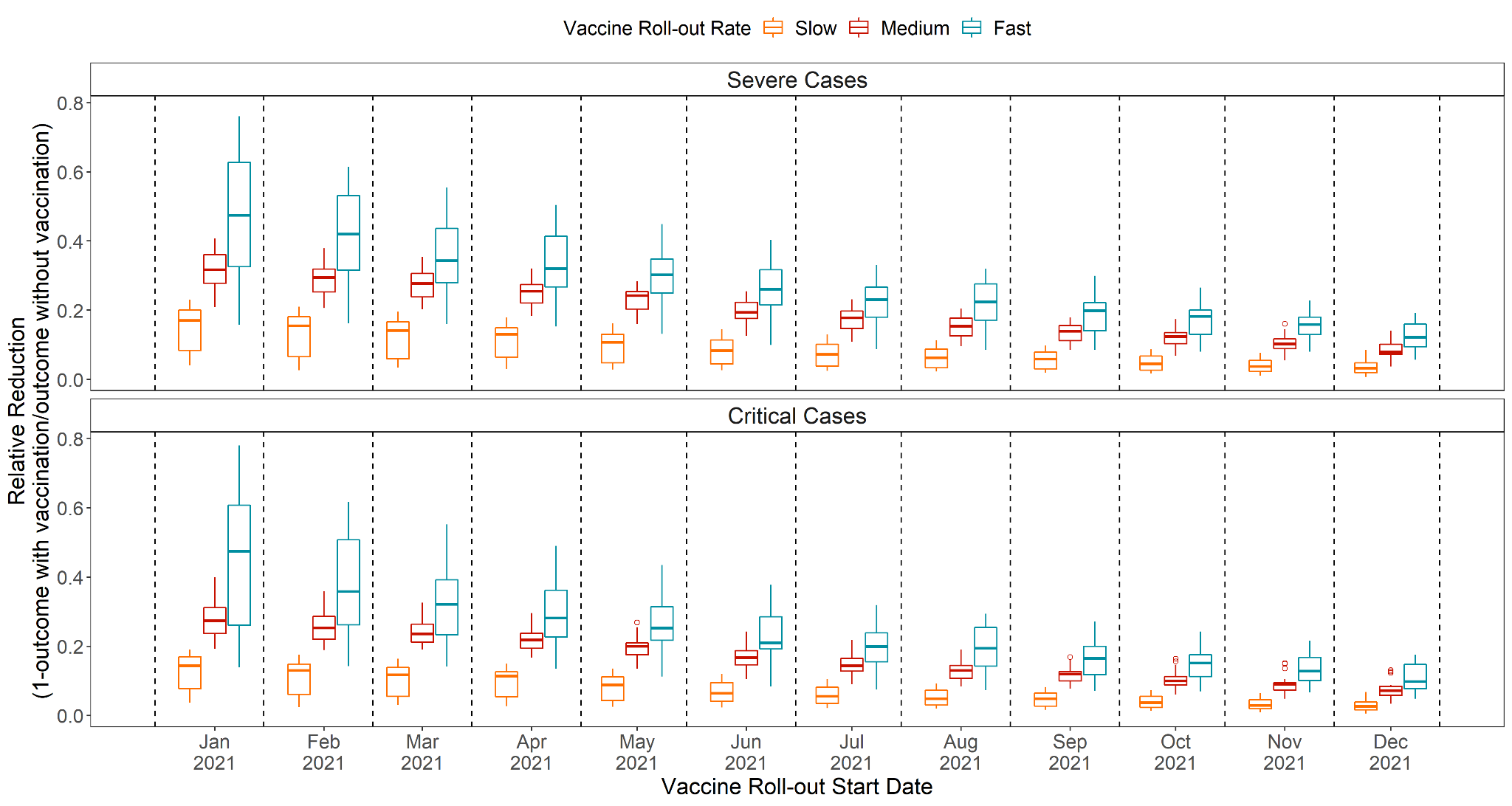


**Caption:** Relative reduction in health burden as a result of different vaccine roll-out scenarios (i.e. combinations of vaccination program start dates and vaccine roll-out rates) for 27 African Union member states. Relative reduction is defined as: 1 - outcome with vaccination/outcome without vaccination. Greater relative reductions indicate more effective vaccine roll-out scenarios, and vice versa.

### Figure S9. Health Outcomes Associated with Different Vaccine Roll-out Scenarios for 27 African Union Members (mRNA vaccines, cases and deaths)


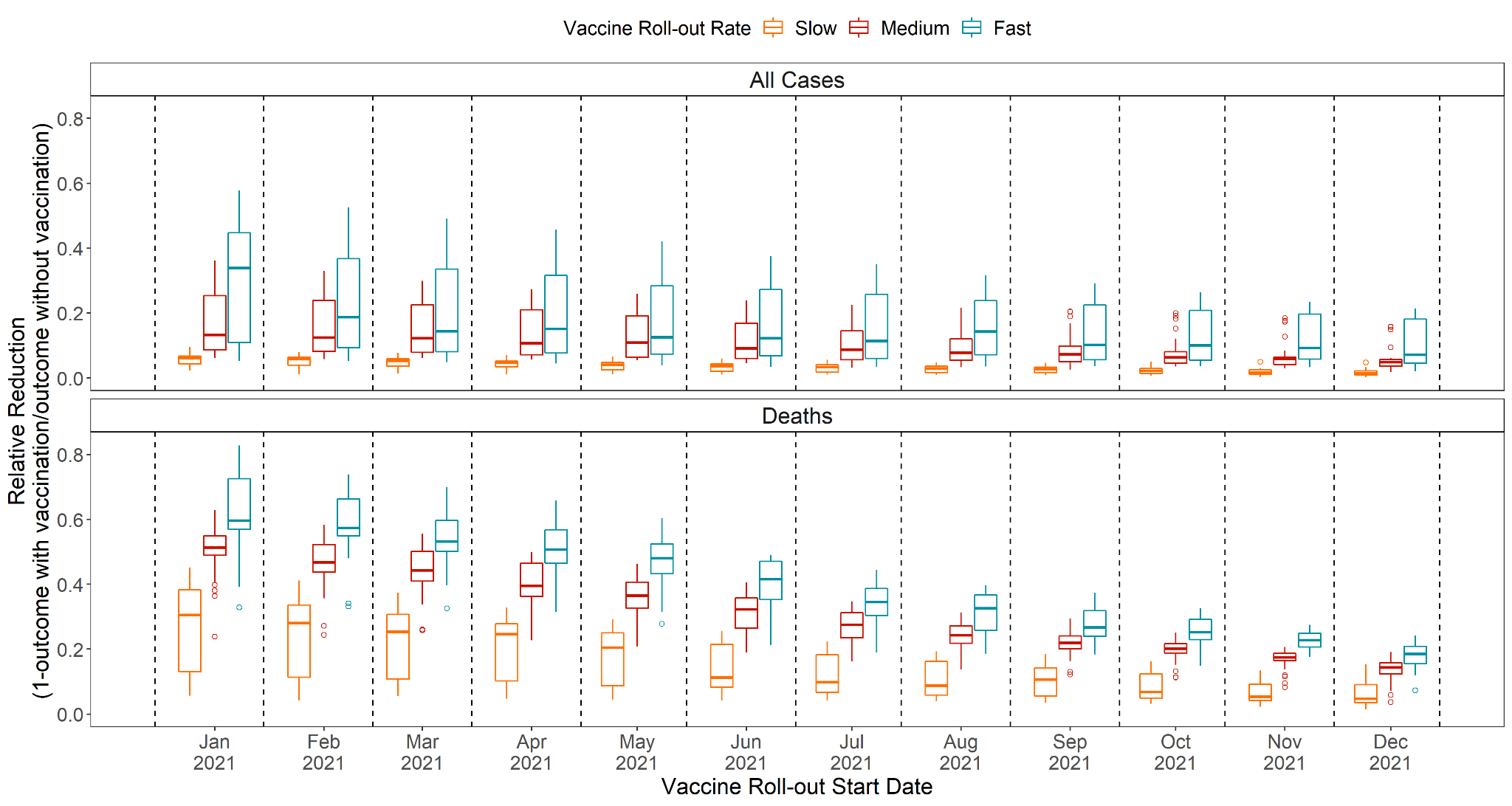


**Caption:** Relative reduction in health burden as a result of different vaccine roll-out scenarios (i.e. combinations of vaccination program start dates and vaccine roll-out rates) for 27 African Union member states. Relative reduction is defined as (1 - outcome with vaccination/outcome without vaccination). Greater relative reductions indicate more effective vaccine roll-out scenarios, and vice versa.

### Figure S10. Health Outcomes Associated with Different Vaccine Roll-out Scenarios for 27 African Union Members (mRNA vaccines, severe and critical cases)


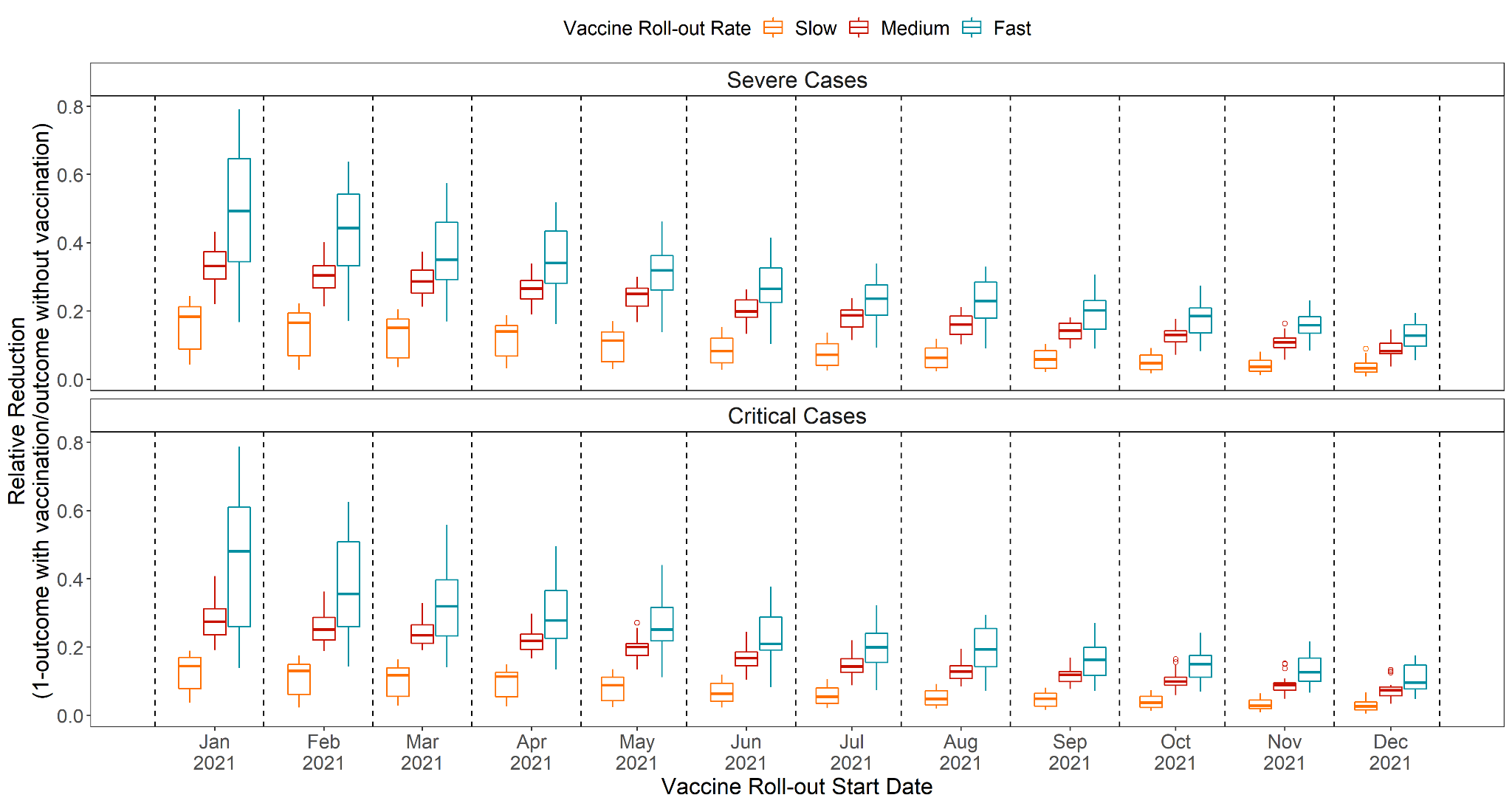


**Caption:** Relative reduction in health burden as a result of different vaccine roll-out scenarios (i.e. combinations of vaccination program start dates and vaccine roll-out rates) for 27 African Union member states. Relative reduction is defined as (1 - outcome with vaccination/outcome without vaccination). Greater relative reductions indicate more effective vaccine roll-out scenarios, and vice versa.

### Figure S11. The association between the proportion of DALYs averted attributable to older adults and the performance of medium and fast scenarios


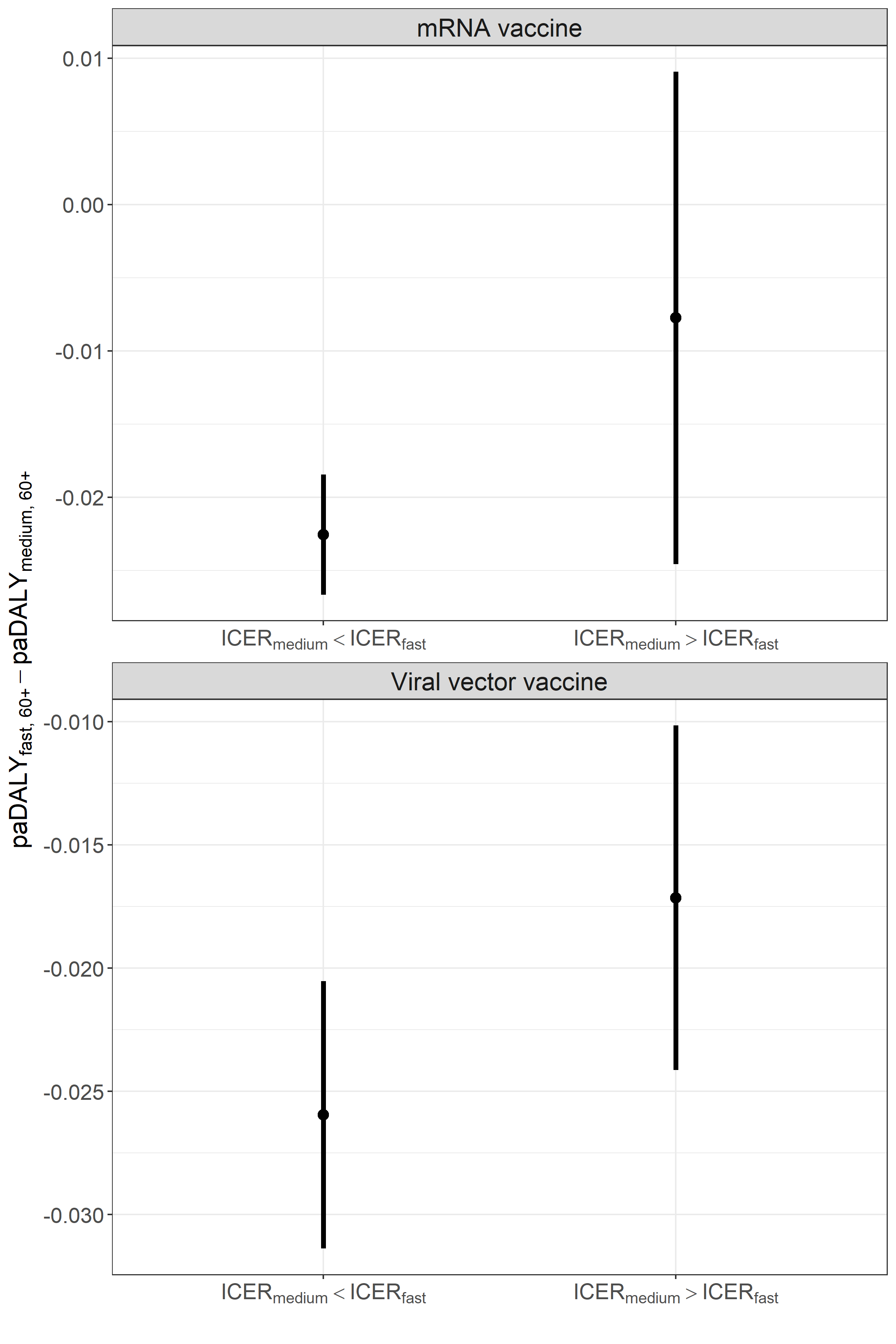


**Caption:** Proportion of DALYs averted (paDALYs) attributed to older adults under medium and fast scenarios were denoted using paDALY_fast, 60+_ and paDALY_medium, 60+_. The differences in means between the two samples (along the x-axis) and the statistical significance of these differences were obtained using two-tailed t-tests. This figure indicates that lower ICERs through relatively slower vaccine roll-out programs are possible due to a higher return on investment in vaccines among older adults.

### Figure S11. Effect sizes estimated in the multi-variable regression model linking country characteristics to ICERs as proportions of GDP per capita


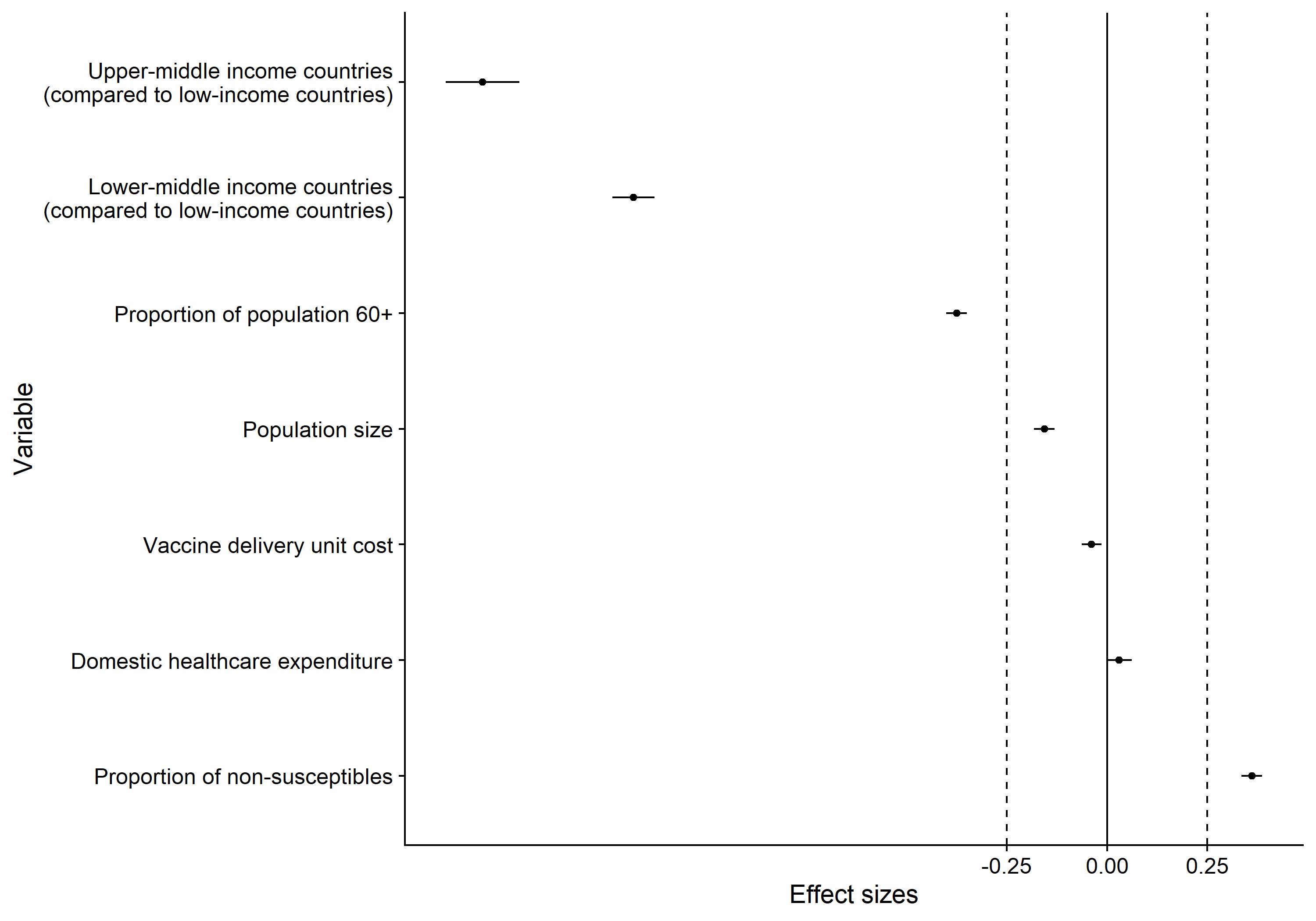


**Caption:** The effect sizes are calculated using a multi-variable linear regression model specified as the following: ICERs as proportions of GDP per capita ~ population size + proportions of population 60 and above + proportion of population non-susceptible at the start of vaccination programmes + income group + domestic healthcare expenditure. Further adjustments have been made to account for vaccine type (mRNA vs. viral vaccine vaccines). All continuous variables have been rescaled for all effect sizes to be on comparable scales. As ICERs as proportions of GDP per capita are calculated for a period, we only tested independent variables that can be summarised for that period. Time-sensitive variables such as non-pharmaceutical interventions that cannot be averaged or summed were excluded. Before the multi-variable analysis, univariable analyses were used to confirm statistical association. We calculated the variance inflation factor to detect potential multi-collinearity issues and found none (all VIF estimates smaller than 3). The relative ranking of effect sizes is robust to further adjustment of vaccine roll-out rates and vaccination programme starting dates.

### Figure S12. Target vaccine price under different perceived cost-effectiveness thresholds


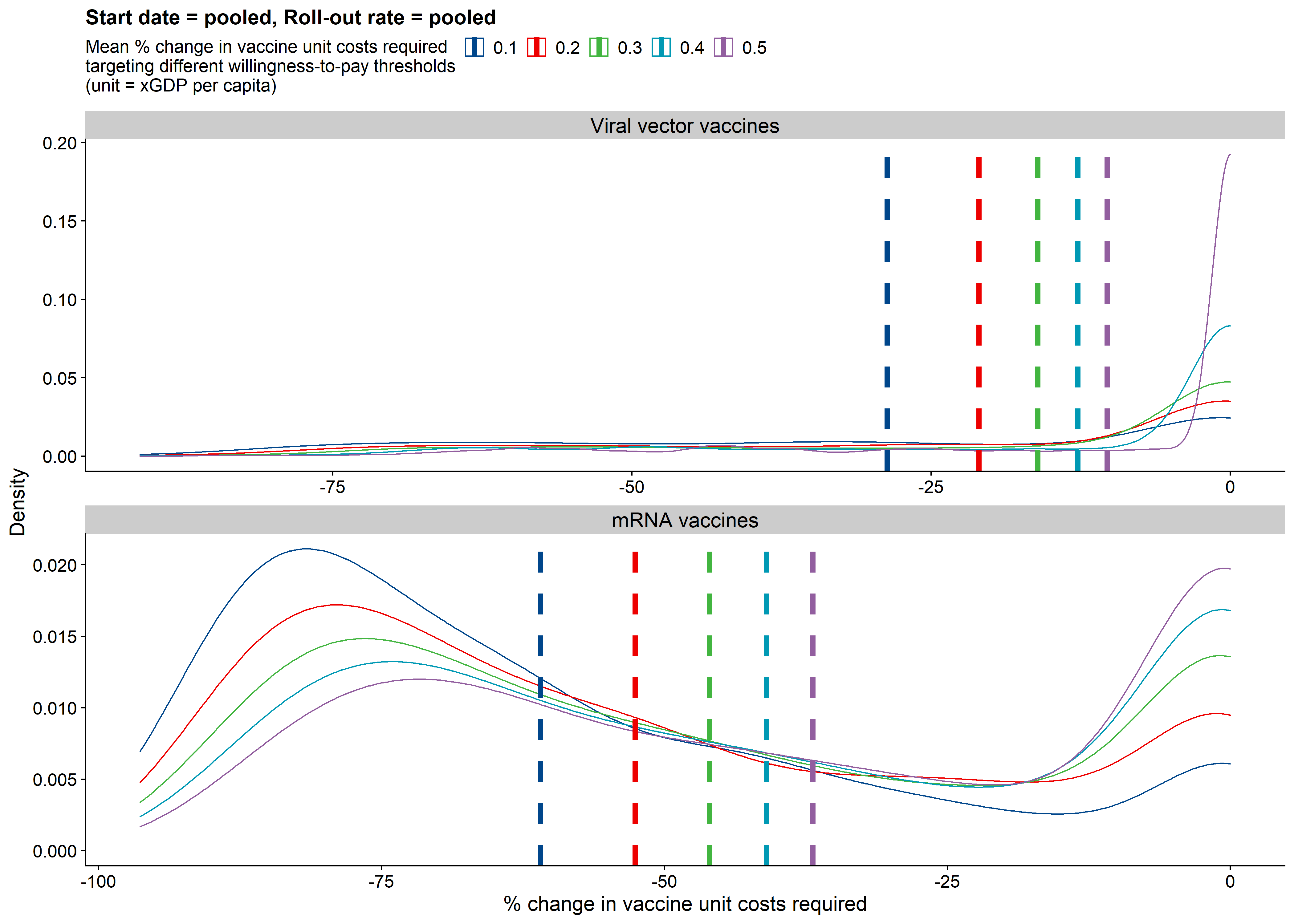


**Caption**: This figure shows the percentage reduction in vaccine unit costs required to reach willingness-to-pay thresholds. Due to the nonmarginal budget impact and the uncertainty around willingness-to-pay thresholds of a Disability Adjusted Life Year, we tested five different potential thresholds: 0.1, 0.2, 0.3, 0.4, and 0.5. Each probability density function (indicated by solid lines) represents 972 data points (27 countries x 36 vaccine roll-out scenarios). The vertical dashed line indicates the mean estimates of these probability density functions. For example, using a willingness-to-pay threshold of 0.1xGDP per capita, the vaccine unit cost of viral vector vaccines needs to be reduced by 28.7% and mRNA vaccines by 61.0% for vaccination programmes to achieve cost-effectiveness on average (across country and vaccine roll-out scenarios.

## Supplemental Tables

### Table S1. Model Equations

| $S_{i}(t)$ | Susceptible individuals among age group $i$ at time $t$ |  |
| --- | --- | --- |
|  | $S_{i}(t + 1)=S_{i}(t)\cdot{(1-\lambda}_{i}(t)) - v_{1i}(t+1)\cdot\frac{S_{i}(t)}{P_{1i}(t)} +R_{i}(t)\cdot\omega_{n}$ | [a] |
| $V_{1i}(t)$ | Individuals among age group $i$ who received their first doses at time $t$ |  |
|  | $V_{1i}(t+1)={V_{1i}(t)\cdot(1-\lambda_{i}(t)\cdot(1-{ve}_{i}) -\omega_{v}) + v}_{1i}(t+1)\cdot\frac{S_{i}(t)}{P_{1i}(t)}-$  $v_{2i}(t+1)\cdot\frac{V_{1i}(t)}{P_{2i}(t)}$ | [b] |
| $S_{wi}(t)$ | Individuals among age group $i$ who received their first doses but the protection has waned at time $t$ |  |
|  | $S_{wi}\left( t+1 \right)={S_{wi}(t)\cdot{(1-\lambda}_{i}(t))+V}_{1i}\left( t \right)\cdot\omega_{v}- v_{2i}(t+1)\cdot\frac{S_{wi}(t)}{P_{2i}(t)}$ | [c] |
| $V_{2i}(t)$ | Individuals among age group $i$ who received their second doses at time $t$ |  |
|  | $V_{2i}\left( t+1 \right)=V_{2i}\left( t \right)\cdot(1- \lambda_{i}\left( t \right)\cdot\left( 1-{v2e}_{i} \right))+$  $v_{2i}\left( t+1 \right)\cdot(\frac{S_{wi}\left( t \right)}{P_{2i}\left( t \right)} + \frac{V_{1i}\left( t \right)}{P_{2i}\left( t \right)})$ | [d] |
| $E_{i}(t)$ | Exposed individuals who are not protected by vaccines (either unvaccinated or have their first doses already waned) among age group $i$ at time $t$ |  |
|  | $E_{i}\left( t+1 \right)=E_{i}\left( t \right)\cdot(1- \sigma)+S_{i}(t)\cdot\lambda_{i}(t)+S_{wi}(t)\cdot\lambda_{i}(t)$ | [e] |
| $E_{vi}(t)$ | Exposed individuals who are protected by one dose of the vaccine among age group $i$ at time $t$ |  |
|  | $E_{vi}\left( t+1 \right)=E_{vi}\left( t \right)\cdot\left( 1-\sigma\right)+V_{1i}\left( t \right)\cdot\lambda_{i}\left( t \right)\cdot\left( 1-{ve}_{i} \right)$ | [f] |
| $E_{v2i}(t)$ | Exposed individuals who are protected by one dose of the vaccine among age group $i$ at time $t$ |  |
|  | $E_{v2i}(t+1)={E_{v2i}\left( t \right)\cdot\left( 1-\sigma\right)+V}_{2i}(t)\cdot\lambda_{i}(t)\cdot(1-{v2e}_{i})$ | [g] |
| $I_{pi}(t)$ | Pre-clinical infectious individuals among age group $i$ at time $t$ |  |
|  | $I_{pi}(t+1)=I_{pi}(t)\cdot{(1-\gamma}_{p})+E_{i}(t) \cdot\sigma\cdot y_{i} +$  $E_{vi}(t) \cdot\sigma\cdot y_{i} \cdot(1-{ve}_{d}) + E_{v2i}(t) \cdot\sigma\cdot y_{i} \cdot(1-{v2e}_{d})$ | [h] |
| $I_{ci}(t)$ | Clinical infectious individuals among age group $i$ at time $t$ |  |
|  | $I_{ci}(t+1)=I_{ci}(t) \cdot\left( 1-\gamma_{c} \right)+I_{pi}(t)\cdot\gamma_{p}$ | [i] |
| $I_{si}(t)$ | Subclinical infectious individuals among age group $i$ at time $t$ |  |
|  | $I_{si}(t+1)=I_{si}(t)\cdot{(1-\gamma}_{s})+ E_{i}(t) \cdot\sigma\cdot{(1-y}_{i}) +$  $E_{vi}(t) \cdot\sigma\cdot{(1 - y}_{i} \cdot(1-{ve}_{d})) +$  $E_{v2i}(t) \cdot\sigma\cdot{(1 - y}_{i} \cdot(1-{v2e}_{d}))$ | [j] |
| $R_{i}(t)$ | Recovered individuals |  |
|  | $R_{i}\left( t+1 \right)=R_{i}\left( t \right)\cdot\left( 1-\omega_{n} \right)+ I_{si}(t)\cdot\gamma_{s} +I_{ci}(t) \cdot\gamma_{c} -$  $v_{1i}(t+1)\cdot\frac{R_{i}(t)}{P_{1i}}$ | [k] |
| $R_{vi}(t)$ | Individuals who have recovered from their previous infections and have received one dose |  |
|  | $R_{vi}\left( t+1 \right)=R_{vi}\left( t \right)+v_{1i}(t+1)\cdot\frac{R_{i}(t)}{P_{1i}}-v_{2i}(t)\cdot\frac{R_{vi}(t)}{P_{2i}(t)}$ | [l] |
| $R_{v2i}(t)$ | Individuals who have recovered from their previous infections and have received two doses |  |
|  | $R_{v2i}(t+1)=R_{v2i}\left( t \right)+v_{2i}(t)\cdot\frac{R_{v2i}(t)}{P_{2i}(t)}$ | [m] |

(continues to the next page)

In which:

| $\lambda_{i}(t)$ | Is the force of infection on the population $i$ at time $t$:  $\lambda_{i}(t) = u_{i}\cdot\sum_{j = 1}^{J} C_{i,j,t}\cdot\frac{(I_{pj}(t) + I_{cj}(t) + I_{sj}(t)\cdot f)}{N_{j}}$  Where $j$ depicts age group, $J$ is 16, $f$ is the relative infectiousness of subclinical individuals compared to pre-clinical and clinical individuals (i.e. 50%), and $u_{i}$ is susceptibility. |
| --- | --- |
| $P_{1i}(t)$ | Is the population eligible for the first doses in the population  $i$ at time $t$:  $P_{1i}(t) = N_{i} -(V_{1i}(t) + S_{wi}(t) + R_{vi}(t) + E_{vi}(t)) -{(R}_{v2i}(t)+V_{2i}(t))$ |
| $P_{2i}(t)$ | Is the population eligible for the second doses in the population  $i$ at time $t$:  $P_{2i}(t) =V_{1i}(t) + S_{wi}(t) + R_{vi}(t) + E_{vi}(t)$ |
| $v_{1i}(t)$ | Is the number of doses to vaccinate the population with dose 1, this is pre-calculated based on vaccine dosing interval strategies. |
| $v_{2i}(t)$ | Is the number of doses to vaccinate the population with dose 2, this is pre-calculated based on vaccine dosing interval strategies. |

**Caption:** This pair of tables has previously appeared in Liu et al. [(1)](https://sciwheel.com/work/citation?ids=12801904&pre=&suf=&sa=0)

###

### Table S2. Epidemic and Healthcare Process Parameters*

| **Parameter** | **Value** | **Source** |
| --- | --- | --- |
| Age-specific susceptibility ($u_{i}$) | 0.38 - 0.88 | Davies et al.[(3)](https://sciwheel.com/work/citation?ids=9102939&pre=&suf=&sa=0) |
| Age-specific clinical progression rates ($y_{i}$) | 0.21 - 0.70 | Davies et al[(3)](https://sciwheel.com/work/citation?ids=9102939&pre=&suf=&sa=0) |
| Age-specific infection fatality rates | ^Raw input: 5.2e-6 - 0.13  By age group: 6.7e-6 – 8.1e-2 | Levin et al.[(4)](https://sciwheel.com/work/citation?ids=10252041&pre=&suf=&sa=0) |
| Age-specific infection severity rates  (hospitalisation) | ^Raw input: 6.3e-4 – 0.17  By age group: 7.2e-4 – 0.13 | Salje et al.  [(5)](https://sciwheel.com/work/citation?ids=8898230&pre=&suf=&sa=0&dbf=0) |
| Age-specific infection critical rates  (ICU) | 1.2e-4 – 1.2e-2 | Salje et al.[(5)](https://sciwheel.com/work/citation?ids=8898230&pre=&suf=&sa=0&dbf=0)  Davies et al.[(6)](https://sciwheel.com/work/citation?ids=10245997&pre=&suf=&sa=0&dbf=0)  Spline fitted using CO-CIN data |
| Age- and country-specific within-population contact pattern  ($C_{i,j,0}$) | Country-specific | Prem et al.[(7)](https://sciwheel.com/work/citation?ids=11703857&pre=&suf=&sa=0) |
| Country-specific population age structures | Country-specific | United Nations[(8)](https://sciwheel.com/work/citation?ids=8436701&pre=&suf=&sa=0&dbf=0) |
| Relationship between mobility and population contact pattern | Defined by linear and nonlinear functions for the *workplace* and *other* settings, respectively. | Davies et al. by fitting to UK data[(6)](https://sciwheel.com/work/citation?ids=10245997&pre=&suf=&sa=0) |
| Latent period ($1/\sigma$) | ~gamma (μ = 2.5, k = 2.5) | Pearson et al.[(9)](https://sciwheel.com/work/citation?ids=12468223&pre=&suf=&sa=0&dbf=0)  Davies et al.[(10)](https://sciwheel.com/work/citation?ids=9011259&pre=&suf=&sa=0)  Davies et al.[(6)](https://sciwheel.com/work/citation?ids=10245997&pre=&suf=&sa=0)  Bi et al.[(11)](https://sciwheel.com/work/citation?ids=8792615&pre=&suf=&sa=0)  Liu et al.[(12)](https://sciwheel.com/work/citation?ids=14346765&pre=&suf=&sa=0&dbf=0)  Linton et al.[(13)](https://sciwheel.com/work/citation?ids=8415907&pre=&suf=&sa=0)  Nishiura et al.[(14)](https://sciwheel.com/work/citation?ids=8415585&pre=&suf=&sa=0) |
| Duration of preclinical infectiousness  ($1/\gamma_{p}$) | ~gamma (μ = 1.5, k = 4) |  |
| Duration of clinical infectiousness  ($1/\gamma_{c}$) | ~gamma (μ = 3.5, k = 4) |  |
| Duration of subclinical infectiousness  ($1/\gamma_{s}$) | ~gamma (μ = 5, k = 4) | Assumed, consistent with Davies et al.[(10)](https://sciwheel.com/work/citation?ids=9011259&pre=&suf=&sa=0) |
| Relative infectiousness of subclinical infections compared to clinical infections  ($f$) | 0.5 | Assumed, consistent with Davies et al.[(10)](https://sciwheel.com/work/citation?ids=9011259&pre=&suf=&sa=0) |
| Duration of infection-induced immunity  (1/$\omega_{n}$) | 3 years | Hall et al.[(15)](https://sciwheel.com/work/citation?ids=14341998&pre=&suf=&sa=0&dbf=0) |
| Proportion of symptomatic cases that require home-based care | 10% | Torres-Rueda et al. [(16)](https://sciwheel.com/work/citation?ids=12767344&pre=&suf=&sa=0) |

This table is similar to that appeared in the supplemental content of Liu et al. [(1)](https://sciwheel.com/work/citation?ids=12801904&pre=&suf=&sa=0)

^ The original studies where these values were extracted had higher age resolution than what we used in the transmission model. Therefore, both the range of the raw data and the range by age group used in this study are provided for context.

###

### Table S3. Additional Data Sources*

| **Parameter** | **Source** |
| --- | --- |
| Country-level daily COVID-19 Mortality (including 7-day rolling average) | Ritchie et al.[(2)](https://sciwheel.com/work/citation?ids=11703906&pre=&suf=&sa=0) |
| Country-level daily COVID-19 vaccine uptake | Ritchie et al.[(2)](https://sciwheel.com/work/citation?ids=11703906&pre=&suf=&sa=0) |
| Observed country-specific community mobility | Google[(17)](https://sciwheel.com/work/citation?ids=11703648&pre=&suf=&sa=0) |
| COVID-19 Government Response Stringency Index and Government Response Tracker by country | Hale et al.[(18)](https://sciwheel.com/work/citation?ids=10678231&pre=&suf=&sa=0) |
| Gross domestic product per capita | World Bank [(19)](https://sciwheel.com/work/citation?ids=13581342&pre=&suf=&sa=0&dbf=0) |
| Annual government general health expenditure | World Bank [(20)](https://sciwheel.com/work/citation?ids=12819804&pre=&suf=&sa=0&dbf=0) |
| Income groups | World Bank  [(21)](https://sciwheel.com/work/citation?ids=13585585&pre=&suf=&sa=0&dbf=0) |

* Part of this table has previously appeared in the supplemental content of Liu et al. [(22)](https://sciwheel.com/work/citation?ids=12467362&pre=&suf=&sa=0&dbf=0)

### Table S4. Other vaccine and vaccination program characteristics

| **Parameter** | **Value** | **Source** |
| --- | --- | --- |
| Supply delay | 4 weeks | Assumed |
| Dosing interval | 4 weeks | WHO [(23)](https://sciwheel.com/work/citation?ids=12766627&pre=&suf=&sa=0)  UK HSA [(24)](https://sciwheel.com/work/citation?ids=12766626&pre=&suf=&sa=0) |
| First dose protection duration (1/$\omega_{v}$) | 360 days | Assumed |
| Age-specific Prioritisation | 60+ year-olds are prioritised over 20-59. In other words, vaccination among those between 20 and 59 years does not start until the coverage cap among 60+ years has been met. | Liu et al. [(22)](https://sciwheel.com/work/citation?ids=12467362&pre=&suf=&sa=0)  based on the vaccine efficacy estimates assumed in this study.  WHO [(25)](https://sciwheel.com/work/citation?ids=13556792&pre=&suf=&sa=0&dbf=0)  UNICEF[(26)](https://sciwheel.com/work/citation?ids=13556794&pre=&suf=&sa=0&dbf=0) |
| Maximum vaccine supply at the population level | 0.7 | WHO [(27)](https://sciwheel.com/work/citation?ids=12801839&pre=&suf=&sa=0) |
| Maximum willingness to receive vaccination by age group | 0.6 for those between 20-59 and 0.8 for those above 60 | Assumed based on Robinson et al.[(28)](https://sciwheel.com/work/citation?ids=11230457&pre=&suf=&sa=0) |

###

### Table S5. Variants of Concern Introduction

| Variants of Concern (VOC) Index | Introduction Date | Single VOC Introduction Countries  (n = 24) | | | | | |
| --- | --- | --- | --- | --- | --- | --- | --- |
|  |  | Transmissibility | | Severity | | Vaccine Effectiveness/ Efficacy  (Against Infection and Disease) | |
|  |  | Step Change | Overall change | Step Change | Overall change | Step Change | Overall change |
| VOC_1 | - | 1 | 1 | 1 | 1 | 1 | 1 |
| VOC_2 | Fitted | 1.5 | 1.5 | 1.5 | 1.5 | 0.8 | 0.8 |
| VOC_3 | 01/12/2021 | 2.25 | **3.375** | 0.5 | **0.75** | 0.7 | **0.56** |
|  | | | | | | | |
| VOC Index | Introduction Date | Double VOC Introduction Countries  (n = 3) | | | | | |
|  |  | Transmissibility | | Severity | | Vaccine Effectiveness/ Efficacy  (Against Infection and Disease) | |
|  |  | Step Change | Overall change | Step Change | Overall change | Step Change | Overall change |
| VOC_1 | Fitted | 1.5 | 1.5 | 1 | 1 | 1 | 1 |
| VOC_2 | Fitted | 1.5 | 2.25 | 1.5 | 1.5 | 0.8 | 0.8 |
| VOC_3 | 01/12/2021 | 1.5 | **3.375** | 0.5 | **0.75** | 0.7 | **0.56** |

Caption: VOC_3 is modelled after the Omicron variant. VOC_1 and VOC_2 are pre-Omicron and post-wild type strains. The transmissibility of the pre-Omicron variants is assumed based on evidence in Barnard et al.[(29)](https://sciwheel.com/work/citation?ids=13511412&pre=&suf=&sa=0&dbf=0) The transmissibility of the Omicron variant was assumed based on evidence in Pearson et al.[(30)](https://sciwheel.com/work/citation?ids=12346822&pre=&suf=&sa=0) The severity of pre-Omicron variants is assumed based on Grint et al. and a Public Health England report. [(31,32)](https://sciwheel.com/work/citation?ids=12936474,12936483&pre=&pre=&suf=&suf=&sa=0,0) The severity of the Omicron variant was assumed based on Danielle Iuliano et al and a UKHSA report. [(33,34)](https://sciwheel.com/work/citation?ids=12936429,12350278&pre=&pre=&suf=&suf=&sa=0,0) The immune-evasiveness of pre-Omicron variants is based on Pouwels et al. and a UK HSA report. [(35,36)](https://sciwheel.com/work/citation?ids=11880224,12936505&pre=&pre=&suf=&suf=&sa=0,0) The immune-evasiveness of the Omicron variant is assumed based on Pearson et al. [(30)](https://sciwheel.com/work/citation?ids=12346822&pre=&suf=&sa=0)

### Table S6. List of countries with fitted models

| Country Name | ISO3 Code | Fitting Window Starting Date | Fitting Window Ending Date |
| --- | --- | --- | --- |
| Algeria | DZA | 25/02/2020 | 12/09/2021 |
| Angola | AGO | 20/03/2020 | 02/11/2021 |
| Botswana | BWA | 30/03/2020 | 01/09/2021 |
| Cameroon | CMR | 06/03/2020 | 11/01/2022 |
| Dem. Republic of the Congo | COD | 11/03/2020 | 11/01/2022 |
| Egypt | EGY | 14/02/2020 | 17/10/2021 |
| Eswatini | SWZ | 14/03/2020 | 22/09/2021 |
| **Ethiopia** | **ETH** | **13/03/2020** | **11/01/2022** |
| **Ghana** | **GHA** | **14/03/2020** | **23/12/2021** |
| Kenya | KEN | 06/03/2020 | 11/01/2022 |
| Liberia | LBR | 17/03/2020 | 07/01/2022 |
| Libya | LBY | 04/03/2020 | 15/09/2021 |
| Madagascar | MDG | 20/03/2020 | 11/01/2022 |
| Malawi | MWI | 29/03/2020 | 11/01/2022 |
| Morocco | MAR | 07/02/2020 | 26/03/2021 |
| Mozambique | MOZ | 22/03/2020 | 04/11/2021 |
| Namibia | NAM | 14/03/2020 | 21/10/2021 |
| **Nigeria** | **NGA** | **28/02/2020** | **11/01/2022** |
| Rwanda | RWA | 14/03/2020 | 10/09/2021 |
| Senegal | SEN | 28/02/2020 | 11/01/2022 |
| Somalia | SOM | 16/03/2020 | 11/01/2022 |
| South Africa | ZAF | 07/02/2020 | 27/08/2021 |
| Sudan | SDN | 13/03/2020 | 11/01/2022 |
| Tunisia | TUN | 04/03/2020 | 18/07/2021 |
| Uganda | UGA | 21/03/2020 | 20/12/2021 |
| Zambia | ZMB | 18/03/2020 | 11/01/2022 |
| Zimbabwe | ZWE | 20/03/2020 | 10/08/2021 |

Caption: Bold font indicates countries with two variants of concern introduction dates fitted pre-Omicron. All other countries only had one variant of concern introduction.

### Table S7. Itemised cost per dose per activity for base countries - viral vector / AstraZeneca-like vaccine - USD$ 2021

| **Activity** | **Ethiopia** | **Nigeria** | **South Africa** |
| --- | --- | --- | --- |
| Planning and coordination | 0.21 | 0.58 | 0.58 |
| Technical Assistance | 0.02 | 0.02 | 0.07 |
| Training | 0.12 | 0.05 | 0.02 |
| Social mobilization | 0.43 | 0.32 | 0.22 |
| Vaccine transport | 0.08 | 0.05 | 0.28 |
| Cold chain | 0.32 | 0.62 | 0.54 |
| Personal protective equipment | 0.24 | 0.18 | 0.09 |
| Hand hygiene | 0.02 | 0.08 | 0.12 |
| Vaccine dose | 3.93 | 3.93 | 3.93 |
| Vaccine delivery | 0.49 | 0.28 | 0.60 |
| Vaccination certificates | 0.15 | 0.34 | 0.28 |
| Waste management | 0.06 | 0.05 | 0.06 |
| Pharmacovigilance | 0.08 | 0.02 | 0.18 |
| **Unit cost per dose**^a^ | **$ 6.16** | **$ 6.52** | **$ 6.96** |

^a^ Facility-based delivery for 12 months campaign based on country coverage targets

###

### Table S8. Itemised Cost per dose per activity for base countries - mRNA / Pfizer-like vaccine - USD$ 2021

| **Activity** | **Ethiopia** | **Nigeria** | **South Africa** |
| --- | --- | --- | --- |
| Planning and coordination | 0.21 | 0.58 | 0.58 |
| Technical Assistance | 0.02 | 0.02 | 0.07 |
| Training | 0.12 | 0.05 | 0.02 |
| Social mobilization | 0.43 | 0.32 | 0.22 |
| Vaccine transport | 0.03 | 0.05 | 0.28 |
| Cold chain | 0.33 | 0.71 | 0.54 |
| Personal protective equipment | 0.24 | 0.18 | 0.09 |
| Hand hygiene | 0.02 | 0.08 | 0.12 |
| Vaccine dose | 13.09 | 13.09 | 13.09 |
| Vaccine delivery | 0.49 | 0.28 | 0.60 |
| Vaccination certificates | 0.15 | 0.34 | 0.28 |
| Waste management | 0.06 | 0.05 | 0.06 |
| Pharmacovigilance | 0.08 | 0.02 | 0.18 |
| **Unit cost per dose**^a^ | **15.29** | **15.77** | **16.13** |

^a^ Facility-based delivery for 12 months campaign based on country coverage targets

###

### Table S9. Cost per dose for countries with fitted models - USD$ 2021

| **Country Name** | **ISO3 Code** | **Viral vector vaccines** | **mRNA vaccines** |
| --- | --- | --- | --- |
| Angola | AGO | 7.15 | 16.39 |
| Botswana | BWA | 8.48 | 17.71 |
| Cameroon | CMR | 5.45 | 14.68 |
| Congo - Kinshasa | COD | 6.81 | 16.05 |
| Algeria | DZA | 15.38 | 24.61 |
| Egypt | EGY | 12.68 | 21.91 |
| Ethiopia | ETH | 6.23 | 15.46 |
| Ghana | GHA | 7.02 | 16.25 |
| Kenya | KEN | 11.95 | 21.18 |
| Liberia | LBR | 5.51 | 14.74 |
| Libya | LBY | 14.30 | 23.53 |
| Morocco | MAR | 7.96 | 17.19 |
| Madagascar | MDG | 6.25 | 15.48 |
| Mozambique | MOZ | 5.27 | 14.50 |
| Malawi | MWI | 5.36 | 14.59 |
| Namibia | NAM | 8.37 | 17.60 |
| Nigeria | NGA | 6.90 | 16.13 |
| Rwanda | RWA | 6.05 | 15.28 |
| Sudan | SDN | 6.71 | 15.94 |
| Senegal | SEN | 4.94 | 14.17 |
| Somalia | SOM | 5.38 | 14.61 |
| Eswatini | SWZ | 7.86 | 17.09 |
| Tunisia | TUN | 7.57 | 16.80 |
| Uganda | UGA | 8.75 | 17.98 |
| South Africa | ZAF | 13.83 | 23.06 |
| Zambia | ZMB | 5.99 | 15.22 |
| Zimbabwe | ZWE | 6.85 | 16.08 |

Caption: This is based on facility-based vaccine delivery, medium vaccine roll-out rates and 18-month vaccination campaign duration. These values correspond to the main text Figure 1e.

### Table S10. Consolidated Health Economic Evaluation Reporting Standards (CHEERS) 2022 Checklist

| **Topic** | **No.** | **Item** | **Location where item is reported** |
| --- | --- | --- | --- |
| Title |  |  |  |
|  | 1 | Identify the study as an economic evaluation and specify the interventions being compared. | **1** |
| Abstract |  |  |  |
|  | 2 | Provide a structured summary that highlights context, key methods, results, and alternative analyses. | **2** |
| Introduction |  |  |  |
| Background and objectives | 3 | Give the context for the study, the study question, and its practical relevance for decision making in policy or practice. | **3** |
| Methods |  |  |  |
| Health economic analysis plan | 4 | Indicate whether a health economic analysis plan was developed and where available. | **n/a**  **Not trial based** |
| Study population | 5 | Describe characteristics of the study population (such as age range, demographics, socioeconomic, or clinical characteristics). | **3-6, Additional File 1: Table S2-S8** |
| Setting and location | 6 | Provide relevant contextual information that may influence findings. | **4-10** |
| Comparators | 7 | Describe the interventions or strategies being compared and why chosen. | **4-5** |
| Perspective | 8 | State the perspective(s) adopted by the study and why chosen. | **4, 8, 9, 17, 18** |
| Time horizon | 9 | State the time horizon for the study and why appropriate. | **7, 9** |
| Discount rate | 10 | Report the discount rate(s) and reason chosen. | **7, 9** |
| Selection of outcomes | 11 | Describe what outcomes were used as the measure(s) of benefit(s) and harm(s). | **6-10** |
| Measurement of outcomes | 12 | Describe how outcomes used to capture benefit(s) and harm(s) were measured. | **6-10**  **Additional File 1: Methods S4** |
| Valuation of outcomes | 13 | Describe the population and methods used to measure and value outcomes. | **6-10** |
| Measurement and valuation of resources and costs | 14 | Describe how costs were valued. | **8-9, Additional File 1: Methods S7-9** |
| Currency, price date, and conversion | 15 | Report the dates of the estimated resource quantities and unit costs, plus the currency and year of conversion. | **8-9**  **Additional File 1: Methods S7-9, Table S7-9** |
| Rationale and description of model | 16 | If modelling is used, describe in detail and why used. Report if the model is publicly available and where it can be accessed. | **4-6, 8-9, Additional File 1: Table S1, Figure S2, Methods S1-4** |
| Analytics and assumptions | 17 | Describe any methods for analysing or statistically transforming data, any extrapolation methods, and approaches for validating any model used. | **4-10**  **Additional File 1: Methods S8-9** |
| Characterising heterogeneity | 18 | Describe any methods used for estimating how the results of the study vary for subgroups. | **4-6**  **Additional File 1: Figure S1 and S11** |
| Characterising distributional effects | 19 | Describe how impacts are distributed across different individuals or adjustments made to reflect priority populations. | **4-5, Additional File 1: Methods S1, S3-6,**  **Figure S11** |
| Characterising uncertainty | 20 | Describe methods to characterise any sources of uncertainty in the analysis. | **6-9** |
| Approach to engagement with patients and others affected by the study | 21 | Describe any approaches to engage patients or service recipients, the general public, communities, or stakeholders (such as clinicians or payers) in the design of the study. | **4, 8-9**  **Additional File 1: Method S7** |
| Results |  |  |  |
| Study parameters | 22 | Report all analytic inputs (such as values, ranges, references) including uncertainty or distributional assumptions. | **4-10**  **Additional File 1: Table S1-6** |
| Summary of main results | 23 | Report the mean values for the main categories of costs and outcomes of interest and summarise them in the most appropriate overall measure. | **10-12, 14-16** |
| Effect of uncertainty | 24 | Describe how uncertainty about analytic judgments, inputs, or projections affect findings. Report the effect of choice of discount rate and time horizon, if applicable. | **10-16**  **Additional File 1: Figures S4-11** |
| Effect of engagement with patients and others affected by the study | 25 | Report on any difference patient/service recipient, general public, community, or stakeholder involvement made to the approach or findings of the study | **n/a, no patient involved** |
| Discussion |  |  |  |
| Study findings, limitations, generalisability, and current knowledge | 26 | Report key findings, limitations, ethical or equity considerations not captured, and how these could affect patients, policy, or practice. | **17-18** |
| Other relevant information |  |  |  |
| Source of funding | 27 | Describe how the study was funded and any role of the funder in the identification, design, conduct, and reporting of the analysis | **21** |
| Conflicts of interest | 28 | Report authors conflicts of interest according to journal or International Committee of Medical Journal Editors requirements. | **23** |

##

From: Husereau D, Drummond M, Augustovski F, et al. Consolidated Health Economic Evaluation Reporting Standards 2022 (CHEERS 2022) Explanation and Elaboration: A Report of the ISPOR CHEERS II Good Practices Task Force. Value Health 2022;25. doi:10.1016/j.jval.2021.10.008

## Supplemental Methods

### Methods S1. Further Model Descriptions

The conceptual diagram of this model can be found in Additional File 1: Fig. S1. The model input parameters are presented in Additional File 1: Table S1-4. The adaptation of CovidM that we are using here has been previously described in detail in Liu et al.[(1)](https://sciwheel.com/work/citation?ids=12801904&pre=&suf=&sa=0) In short, the model has 13 infection-/ disease-related compartments and age stratification into 16 age groups (ranging from 0-4 to 75+ with five-year increments), totally 208 compartments.

Differences between age groups are characterised by age-specific parameters such as contact rates,[(7)](https://sciwheel.com/work/citation?ids=11703857&pre=&suf=&sa=0) susceptibility, clinical fraction,[(3)](https://sciwheel.com/work/citation?ids=9102939&pre=&suf=&sa=0) and infection fatality and hospitalisation rates.[(4,5)](https://sciwheel.com/work/citation?ids=10252041,8898230&pre=&pre=&suf=&suf=&sa=0,0)

Differences between countries in the context of the transmission model are characterised by COVID-19 government response stringency index (SI), Google Mobility Index (GM), population age structure, social contact patterns, daily reported COVID-19 deaths. More specifically, SI and GM are used to modify social contact patterns to capture the behavioural changes during the pandemic essential to the transmission of SARS-CoV-2. More details on the method can be found in the Additional File 1: Methods S3.

Compared to a convention SEIR (susceptible-exposed-infectious-removed) model, this adaptation of CovidM:

- can more accurately capture the natural progression of COVID-19 using the “pre-clinical & infectious” and “subclinical & infectious compartments”;
- allows both susceptible and removed individuals to receive vaccination. Doses given to Individuals during an infection are considered “wasted”;
- allows for the incorporation of six different types of vaccine effect mechanisms (infection-, disease-, severe disease-, critical disease-, mortality-reducing and onward transmission-preventing);
- allows for the introduction of variants of concerns with changes in susceptibility, severity, and immune evasiveness.
- can more accurately capture the changes in behaviour during the pandemic using SI and GM.

In this model, we assumed that infection-induced immunity among individuals who have never received vaccines and immunity over individuals who had breakthrough infections (vaccinated and then infected) would wane exponentially with an average duration of 3 years.[(15)](https://sciwheel.com/work/citation?ids=14341998&pre=&suf=&sa=0&dbf=0) Fully vaccinated individuals without infection history and recovered individuals who then received vaccines do not experience any protection waning effect given this the relatively short time horizon of this study. The lack of waning among recovered individuals who then received vaccines allows us to capture the potential immune enhancement observed in some cases.[(37)](https://sciwheel.com/work/citation?ids=10652101&pre=&suf=&sa=0) This setup also improves our capacity in vaccine dose accounting - making sure that most individuals in this model receive only two doses of vaccines.

###

### Methods S2. Fitting Process

The model fitting methods used in this study have previously been described in Liu et al.

[(1)](https://sciwheel.com/work/citation?ids=12801904&pre=&suf=&sa=0) In brief, we estimated the following parameters by fitting this model to country-level daily reported COVID-19 deaths:

1. the basic reproduction numbers (R0). This is relevant for the wild-type strain circulating starting from late 2019/ early 2020.
2. infection introduction dates. Instead of the first day when a case was reported, this is the first day where infection introduction has led to community transmission.
3. COVID-19 death reporting rate. This is the proportion of COVID-19 deaths reported to the surveillance system. If there are 100 COVID-19 deaths but only 10 deaths were reported, the reporting rate is 10%.
4. Variants of concern introduction dates. We only fitted for pre-Omicron variants of concern introduction dates but not for variants of concern characteristics. The variants of concern characteristics we used are based on evidence from the existing literature (see Additional File 1: Table S5). For 24 countries, one variant of concern introduction would allow us to capture the epidemic waves. For 3 countries (Ethiopia, Ghana, and Nigeria), two variants of concern introductions were necessary. The choices between one or two variants of concern introductions were based on visual examinations of the epidemic peaks captured.

The model was fitted using differential evolution algorithms, implemented using the `DEoptim` package in R.[(38)](https://sciwheel.com/work/citation?ids=12444549&pre=&suf=&sa=0) We assume daily reported COVID-19 deaths to follow a Poisson distribution.

###

### Methods S3. Characterising behavioural change using data on non-pharmaceutical intervention and mobility

In two previous studies, [(1,22)](https://sciwheel.com/work/citation?ids=12467362,12801904&pre=&pre=&suf=&suf=&sa=0,0) we described how we used the Stringency Index (as defined in the context of the Oxford COVID-19 Government Response Tracker) [(18)](https://sciwheel.com/work/citation?ids=10678231&pre=&suf=&sa=0) and the Google Mobility Index [(17)](https://sciwheel.com/work/citation?ids=11703648&pre=&suf=&sa=0) in great detail. In summary and in the context of this study, for population contact patterns in `work` and `others` settings, we assumed:

1. The relationship between contacts and mobility is defined by functions inferred in Davies et al. based on UK data;[(6)](https://sciwheel.com/work/citation?ids=10245997&pre=&suf=&sa=0)
2. The future changes in mobility beyond available data are approximated using a general additive model. Independent variables include day-of-week, country (random effect), an interaction term between day-of-week and mobility type (e.g. grocery, retail, park), Stringency Index (spline), month, and date (spline, to capture slow-varying long-term trend that’s not otherwise captured elsewhere).
3. Stringency Index is not expected to vary beyond available data. This is based on the observed Stringency Index in most countries three months prior to the end of the observed time series. The Stringency Index used for future changes range from 19.44 (The Republic of Namibia) to 76.75 (The Republic of Sudan) (high values indicate high stringency).

### Methods S4. Calculating COVID-19 severe, critical, and death cases

In this study, we projected four health outcomes under different vaccine roll-out scenarios: (a) symptomatic infections; (b) severe cases that require hospitalisation; (c) critical cases that require intensive care unit (ICU) admission; and (d) deaths. The health system parameters that we are using include infection-hospitalisation rate, infection-ICU rate, and infection fatality rate. The values we used for these parameters were estimated in the context of Europe where healthcare resources are considered relatively abundant compared to the rest of the world. For example, the infection-hospitalisation rate is interpreted as the percentage of infections that requires hospital admissions as we assume those who need hospitalisation were admitted. In the context of the African Union, however, the numbers of individuals that require hospitalisations and those who were admitted are unlikely going to align. Due to scarce health care resources, cumulative outcomes (b) and (csind) are likely going to exceed observation.

Below we provide the procedures that we use to calculate these outcomes (VE = vaccine efficacy). The concepts presented here are similar to our previous studies although we have adapted the equations to capture intermediate outcomes.[(1,22)](https://sciwheel.com/work/citation?ids=12467362,12801904&pre=&pre=&suf=&suf=&sa=0,0)

1. The temporal delays between infection and health outcomes are assumed to follow gamma distributions. The probability density function is capped at 60 days and can be expressed as the following:

P_severe = *Gamma*(mean = 8.5, shape = 5) + *Gramma*(mean = 14.6, shape = 5)

P_critical = *Gamma*(mean = 8.5, shape = 5) + *Gramma*(mean = 15.6, shape = 5)

P_death = *Gamma*(mean = 26, shape = 5)

This set of parameters is consistent with that used in Pearson et al. [(9)](https://sciwheel.com/work/citation?ids=12468223&pre=&suf=&sa=0)

1. The outcomes that occur on day *t* due to infection on day *t-d* can thus be expressed as:

Severe(t)_dj = E_j(t-d) * P_severe(d) * IHR +

Ev1_j(t-d) * P_severe(d) * IHR * (1-VE_severe_1) +

Ev2_j(t-d) * P_severe(d) * IHR * (1-VE_severe_2)

Critical(t)_dj = E_j(t-d) * P_critical(d) * IHR +

Ev1_j(t-d) * P_critical(d) * IHR * (1-VE_critical_1) +

Ev2_j(t-d) * P_critical(d) * IHR * (1-VE_critical_2)

Death(t)_dj = E_j(t-d) * P_death(d) * IHR +

Ev1_j(t-d) * P_death(d) * IHR * (1-VE_death_1) +

Ev2_j(t-d) * P_death(d) * IHR * (1-VE_death_2)

where *j* indicates age group.

1. The cumulative outcomes on day *t* can thus be expressed as

Severe(t) = Sum_{j = 1}{J} * Sum_{d = 1}{60} Severe(t)_dj

Critical(t) = Sum_{j = 1}{J} * Sum_{d = 1}{60} Critical(t)_dj

Deaths(t) = Sum_{j = 1}{J} * Sum_{d = 1}{60} Deaths(t)_dj

Note that in this set of formulas we referenced six VE estimates (VE_severe_1, VE_severe_2, VE_critical_1, VE_critical_2, VE_death_1, VE_death_2). They are not values directly from Table 1 and may require further conversion. Only metrics that end with _o are directly observed. Here, we use the vaccine efficacy against death by the first dose as an example - other VEs were converted using the same method.

1. If m deaths were to occur without vaccine, in a population vaccinated with the first dose we should avert m*VE_death_1_o deaths.
2. What has been described in (1) has two components: a. individuals who did not get infected due to vaccination and b. infected individuals whose dead were averted due to vaccination. Component a. can be expressed as m*VE_infection_o. Component b. Can be expressed as m*(1-VE_infection_o)*VE_death_1
3. With these relationships, we have:

m*VE_death_1_o = m*VE_infection_o + m*(1-VE_infection_o)*VE_death_1

With some rearrangement, we have:

VE_death_1 = (VE_death_1_o - VE_infection_o)/(1-VE_infection_o)

### Methods S5. Lengths of Stay (LoSs)

We based our parameters on lengths of stay (LoSs) based on Leclerc et al. [(39)](https://sciwheel.com/work/citation?ids=12767052&pre=&suf=&sa=0)

|  | **Beds** | **n** | **Stage 1** | **Stage 2** | **Stage 3** | **Mean total lengths of stay (days)** |
| --- | --- | --- | --- | --- | --- | --- |
| Bed pathways involving CC | CC | 232 | 10.91 | - | - | 10.91 |
|  | CC, Ward | 2521 | 13.53 | 7.07 | - | 20.75 |
|  | Ward, CC | 183 | 3.39 | 8.77 | - | 12.18 |
|  | Ward, CC, Ward | 3603 | 4.1 | 12.25 | 6.9 | 23.32 |
| Bed pathways that do not involve CC | Ward | 29975 | 9.6 |  |  | 9.60 |

**Table:** Lengths of stay by bed pathway in COCIN. (CC = critical care)

We calculated the weighted mean length of stay amongst all bed pathways involving a stay in a critical care bed:

(23.32*3603+12.18*183+20.75*2521+10.91*232)/(232+2521+183+3603) = 21.58 days.

This value was used in calculating the total DALYs (more specifically the YLDs) associated with critical cases. The mean LoS not involving any critical care bed use (i.e. 9.6 days) was used in calculating the total DALYs (more specifically the YLDs) associated with severe cases.

(section continued on the next page)

We calculated the weighted mean ward stay and weighted CC stay among bed pathways involving a stay in a critical care bed:

In ward -

((4.1+6.9)*3603 + 3.39*183 + 7.07*2521 + 0*232)/(232+2521+183+3603) = 8.88 days

In CC -

(12.25*3603 + 8.77*183 + 13.53*2521 + 10.91*232)/(232+2521+183+3603) = 12.60 days

These values, together with the LoSs for bed pathways not involving any stay in a critical care bed (i.e. 9.6 days), were used in calculating the total healthcare costs associated with critical and severe cases, respectively. As a reminder, in the context of this study, severe cases are defined as those that require hospitalisation; critical cases are defined as those that require critical/ intensive care at some point during their hospital stay.

### Methods S6. Calculating Disability-adjusted Life Years (DALYs)

We calculated DALYs associated with Covid-19 morbidity and mortality as follows:

- We calculated Years of Life Lost (YLLs) for Covid-19 deaths using country-specific standard life tables; [(8)](https://sciwheel.com/work/citation?ids=8436701&pre=&suf=&sa=0&dbf=0)
- For acute morbidity we will use disability weights for different severity of acute infection and for long-covid using the disability weight for chronic fatigue syndrome (see table below), following a similar approach to Wyper et al. [(40)](https://sciwheel.com/work/citation?ids=12433748&pre=&suf=&sa=0)
  - In line with costing assumptions, we assume that 10% of community cases seek healthcare and therefore have symptoms corresponding to the moderate disability weight and that the remaining 90% of cases experience mild symptoms for a duration of 5 days.
  - For hospitalised cases, we apply the severe weight for 9.60 days for those hospitalised without ICU care, and the critical weight for 21.58 days for those hospitalised with a stay in ICU (see section above on LOSs)
  - For long-covid we conservatively assume 20% of patients experience post-acute consequences lasting for 6 months.

Disability weighting used for different health states is summarised in the following table adapted from Wyper et al. [(40)](https://sciwheel.com/work/citation?ids=12433748&pre=&suf=&sa=0)

| **Health state** | **Description** | **Disability weight** | **Source** |
| --- | --- | --- | --- |
| Asymptomatic | Has infection but does not experience any symptoms | Nil |  |
| Mild | Infectious disease, acute, mild: Has a low fever and mild discomfort, but no difficulty with daily activities. | 0.006  (0.002-0.012) | Salomon et al. [(41)](https://sciwheel.com/work/citation?ids=854002&pre=&suf=&sa=0) |
| Moderate  (Community; seeking healthcare assistance) | Infectious disease, acute episode, moderate: Has a fever and aches and feels weak, which causes some difficulty with daily activities. | 0.051 (0.032–0.074) | Salomon et al. [(41)](https://sciwheel.com/work/citation?ids=854002&pre=&suf=&sa=0) |
| Severe (Hospitalised; non-intensive care) | Infectious disease, acute episode, severe: Has a high fever and pain, and feels very weak, which causes great difficulty with daily activities. | 0.133 (0.088–0.190) | Salomon et al. [(41)](https://sciwheel.com/work/citation?ids=854002&pre=&suf=&sa=0) |
| Critical (Hospitalised; intensive care) | Intensive care admission with or without respiratory support. | 0.655 (0.579–0.727) | Haagsma et al. (European Disability Weight Study) [(42)](https://sciwheel.com/work/citation?ids=2171766&pre=&suf=&sa=0) |
| Post-acute consequences | Chronic Fatigue Syndrome: Always tired and easily upset. The person feels pain all over the body and is depressed. | 0.219 (0.148–0.308) | Salomon et al. [(41)](https://sciwheel.com/work/citation?ids=854002&pre=&suf=&sa=0) |

### Methods S7. Estimating Vaccine Delivery Costs

**Estimating unit costs per dose for base countries**

While estimating vaccine unit cost, we covered 12 vaccination activities necessary for the planning, roll-out and delivery of vaccines, as well as the cost of the dose itself. These are planning and coordination, technical assistance, training, social mobilisation, vaccine transport, cold chain, personal protective equipment, hand hygiene, vaccine delivery, vaccination certificates, waste management and pharmacovigilance. The decision to include these 12 activities was based on a model of the costs of delivering the COVID-19 vaccine in the 92 COVAX countries developed by UNICEF.[(43)](https://sciwheel.com/work/citation?ids=12814598&pre=&suf=&sa=0) The price per dose per country was obtained from the Graduate Institute Geneva’s Covid-19 Vaccine Access which tracks publicly available data on agreements to purchase or supply Covid-19 vaccines.[(44)](https://sciwheel.com/work/citation?ids=14347149&pre=&suf=&sa=0&dbf=0)

To estimate the unit cost per dose we used a normative ingredient-based costing approach.[(45)](https://sciwheel.com/work/citation?ids=12814602&pre=&suf=&sa=0) A normative ingredient-based approach implies that we relied on the per-protocol costs instead of observed costs; that we accounted for itemised costs by broad categories. Due to the normative nature of this approach, we were not able to capture wastes and constraints. For this reason, the vaccine unit cost estimates in this study may have been underestimated.

We calculated full economic costs from a health system perspective over several roll-out rates and program duration. We used recent local cost and resource use data from three base countries in Africa: Ethiopia (low-income country (LIC)), Nigeria (lower-middle-income country (lower-MIC)) and South Africa (upper-middle-income country (upper-MIC)). To estimate the resource use and prices of inputs we conducted a literature review, encompassing both peer-reviewed literature and grey literature. An initial description of plausible resource use and a list of prices was prepared and shared with public health experts in-country, who reviewed and validated it through several rounds of exercises to arrive at a final description for each sub-activity.

We provide a list of assumptions for the three countries that we collected data for. The author could be contacted for further information about the specific values used.

| **Planning and coordination** | Each level of administration will require planning and coordination activities. All planning and coordination staff are assumed to be working with laptops. We assume staff salaries for:  **Ethiopia**   - 10 senior government officials at the national level - 10 junior government officials at the national level - 10 senior government officials per region (120 officials in total) - Two junior government officials per zone (230 officials in total) - Two junior government officials per woreda (2,108 officials in total) - One team supervisor per vaccination facility (4,063 supervisors in total) |
| --- | --- |
|  | **Nigeria**   - 20 senior government officials at the national level - 10 senior government officials per state (370 officials in total) - Seven junior government officials per local area (5,418 officials in total) - One team supervisor per facility (2,600 supervisors in total) |
|  | **South Africa**   - 54 senior government officials at the national level - 10 junior government officials at the national level - 25 senior government officials per province (225 officials in total) - Five junior government officials per province (45 officials in total) - 20 government officials per district (1,040 officials in total) - Two junior government officials per local municipality (410 officials in total) - One facility manager per vaccination site (2,335 managers in total) |
| **Technical Assistance** | International consultants provide assistance on all aspects of vaccine rollout, from planning and coordination and social mobilization to cold chain logistics and monitoring and evaluation. We assume all consultants are working with laptops and that teams of consultants require office space in each region. We assume salaries for:  **Ethiopia**   - Eight international consultants for planning and coordination (including assistance with national training) - Eight international consultants for monitoring and evaluation - Eight international consultants for service delivery - Eight international consultants for demand generation and communication - Eight international consultants for cold chain & logistics |
|  | **Nigeria**   - Four consultants for planning and coordination (including assistance with national training) - Two consultants for social mobilization - Eight consultants for cold chain and logistics - Five consultants for monitoring and evaluation - Five consultants for service delivery |
|  | **South Africa**   - Four international consultants for planning and coordination - 17 international consultants for monitoring and evaluation - Five international consultants for service delivery - Four international consultants for demand generation and communication - Two international consultants for cold chain and logistics |
| **Training** | Training is provided in the form of cascade training from national to subnational levels. Assuming bus transport for all participants of training, and hall rental, refreshments, and stationery for each training session. We assume staff salaries and per diems for:  **Ethiopia**   - One national training of trainers (three days of training): Five senior-level government officials and international/local consultants conduct training for teams of junior officials and physicians from each region (five officials per region) - One training per region (two days of training): conducted by five junior officials per region training three coordinators per zone - One training per zone (two days of training): conducted by three coordinators per zone training teams of three coordinators per woreda - One training per woreda (two days of training): conducted by three coordinators per woreda training teams of two coordinators and vaccination team supervisors per facility - One training per facility (One day of training): conducted by team supervisors training two health extension workers and two nurses per facility |
|  | **Nigeria**   - One national training of trainers (three days of training): Five senior-level government officials and four international consultants conduct training for teams of junior-level government officials and physicians from 37 states (three junior officials and three physicians per state) - One training per state (two days of training): conducted by three junior-level government officials training teams of three nurses, two vaccinators, and two record keepers for each of the 774 local government areas - One training per local government area (two days of training): conducted by three nurses training teams of one vaccinator and one record keeper each per health facility |
|  | **South Africa**  Assuming that all vaccination training is performed online. Individuals involved in vaccination in facility sites must do the training for vaccination sites to be approved. Assume the use of laptops for training sessions   - Training for two nurses (actual average from active vaccination sites), one pharmacist and a data collector per vaccination site (five hours of training) - Additional vaccination electronic health record (EVDS) training (two hours of training) |
| **Social mobilisation** | **Ethiopia**   - Two local consultants per region (24 consultants in total) developing the social mobilization strategy and messaging. - Two support staff and five health extension workers per facility to facilitate community awareness and events. - National TV ads: 60-second advertisement aired three times daily for 15 days - National radio ads: 60-second advertisement aired three times daily for 15 days - Flyers: 1,150,000 printed brochures (10% of coverage target) - Regional radio ads: 60-second advertisement aired three times daily for 15 days - Regional TV ads: 60-second advertisement aired three times daily for 15 days - 48 audio-mounted vehicles used for 15 days - Banners and posters at each vaccination site - Advocacy workshops and capacity-building workshops conducted at national, regional, zone, and woreda levels |
|  | **Nigeria**   - One local consultant per state to develop the social mobilization strategy and messaging. - One local worker and one local leader per health facility to facilitate community awareness and events. - National TV ads: 60-second advertisement aired daily for six months - National radio ads: 60-second advertisement aired daily for six months - Flyers: 200 printed flyers per health facility - Local radio ads (in each local area): 60-second advertisement aired daily for one month |
|  | **South Africa**   - 41 members of a ministerial advisory committee on social change, aimed at driving social behaviour change in communities, providing support - 14 members of the Government Communication Information System (GCIS) lead the communication response. - Two GCIS Provincial Directors provide regional support - One councillor per local municipality and two GCIS workers per ward to facilitate community awareness and events - TV and radio ads at the national and provincial level - Flyers and posters at the facility level |
| **Vaccine transport** | We assume full cold storage capacity at the national level for a single shipment of all doses, and we have allocated numbers of doses equally across regions. Storage assumptions:   - Volume per vaccine dose : 3.76cm^3 - Refrigerated truck volume: 30,000L - Hilux double cab pickup volume (truck bed): 1,204L - Refrigerated truck vaccine storage capacity: 1,563 boxes per truck, 3,751,200 doses - Hilux truck vaccine storage capacity: five boxes per truck, 23,920 doses - Vaccine carrier volume (from woreda to facility): 2.7L, 717 doses - Fuel efficiency: 10km/L - Vaccines are picked up from local area refrigerators by motorcycle and delivered to facilities using long-range vaccine carriers (2.7L).   **Ethiopia**   - Five refrigerated trucks (and five drivers) at the national level - 1,493 hilux double cab trucks (and 1,493 drivers) at the zone and woreda levels - Average distance from regional capital to country capital: 554.2km - Average distance from zone capital to regional capital: 315.97km - Average distance from woreda to zone capital: 353.86km - Average distance from health facility to woreda: 34.3km - Average catchment area of each health facility: 278.7km2 - Number of deliveries in the country (to national stores): nine - Number of deliveries per region by refrigerated truck: five (assuming four days for roundtrip delivery) - Number of deliveries per zone by hilux truck: 26 (assuming two days for roundtrip delivery) - Number of deliveries per woreda by hilux truck: 21 (assuming two days for roundtrip delivery) - Number of deliveries per facility by motorcycle: 33 (assuming one day for roundtrip delivery) |
|  | **Nigeria**   - Three refrigerated trucks (and three drivers) at the national level - 1,659 hilux double cab trucks (and 1,659 drivers) at the local area and ward level - Average distance from the state capital to Abuja: 515km - Average distance from local area capital to the state capital: 109km - Average distance from health facility (or ward) to local area capital: 19km - Number of deliveries in the country (to national stores): three - Number of deliveries per state by refrigerated truck: three (assuming four days for delivery) - Number of deliveries per local area by truck: seven (assuming two days for delivery) - Number of deliveries per ward by truck: three (assuming two days for delivery) - Number of deliveries per facility by motorcycle: 98 (assuming one day for delivery) |
|  | **South Africa**   - Two drivers with two refrigerated trucks take the vaccine doses from OR Tambo Airport to Biovac National Warehouse - Distance from the airport to the warehouse is 30 km. - 17 drivers with 17 refrigerated trucks take the vaccine doses from Biovac National Warehouse to provincial distribution depots. - 2,336 drivers with hilux trucks deliver the doses from the provincial warehouses to each vaccination site (2336 vaccination sites in total). Vaccines are transported in large long-range cold boxes - Each driver does 14 deliveries for single-dose vaccines and 21 for double-dose vaccines. - One day for delivery to national warehouse and facilities, four days (return) for delivery from national warehouses to provincial depots - Average distance from Biovac national warehouse to provincial depots: 714 km - Average distance from provincial depots to health facility: 129 km |
| **Cold chain** | We assume office space rented at the national and subnational level  **Ethiopia**   - 6.5 10,000L cold rooms at the national level - One 10,000L cold room per region (12 in total) - One 92L solar direct drive fridge per zone - One 50L solar direct drive fridge in one-quarter of woredas - Each main power refrigerator uses 19,272 kilowatt-hours per year - Each solar direct drive refrigerator uses 12,176.4 kilowatt-hours per year - Each cold room uses 475,668 kilowatt-hours per year   Cold chain and logistics staff:   - 10 senior-level government officials at the national level - 10 junior-level government officials at the national level - 10 senior-level government officials per region (120 officials in total) - 10 junior-level government officials per region (120 officials in total) - Three junior-level government officials per zone (345 officials in total) - One junior-level government official per woreda (1054 officials in total) |
|  | **Nigeria**   - 15 30,000L cold rooms at the national level - One 10,000L cold room per state (37 in total) - One 145L main power fridges per state (37 in total) - One 92L solar direct drive fridge per local area (774 in total) - 0.5 50L solar direct drive fridge per ward (50% of wards have a fridge - 4783 in total) - Each main power refrigerator uses 19,272 kilowatt-hours per year - Each solar direct drive refrigerator uses 12,176.4 kilowatt-hours per year - Each cold room uses 475,668 kilowatt-hours per year   Cold chain and logistics staff:   - National logistics working group: six members at the national level and 37 at the state level - State logistics working group: 10 members per state (370 in total) - Local area logistics working group: three members per local area (2322 in total) - Cold chain teams are composed of one cold chain officer, one logistics officer, and one immunization officer for six zones, 37 states, and 774 local government areas |
|  | **South Africa**   - Three cold rooms (10,000L) at the national level are devoted to COVID-19 vaccine doses at BIOVAC National Warehouse - 18 cold rooms (10,000L) at the provincial level. - Eight ultra-cold storage freezers (528L) for ultra-cold storage vaccines at Biovac National Warehouse - One ultra-cold storage freezer per province - One Solar direct drive Meta Fridge, 50L per vaccination site (2336) - Each main power refrigerator uses 19,272 kilowatt-hours per year - Each solar direct drive refrigerator uses 12,176.4 kilowatt-hours per year - Each cold room uses 475,668 kilowatt-hours per year   Cold chain and logistics staff:   - 30 cold chain professionals at the national level - 15 cold chain professionals at provincial depots (255 in total) - One pharmacist at the facility level |
| **PPE** | Assuming only surgical masks and examination gloves are used: three surgical masks per person per day and 10 pairs of gloves per vaccinator per day. |
| **Hand hygiene** | Hands washed before each dose: we assume 50% of handwashing is done with soap and water, and 50% of handwashing is done with hand sanitiser. Facilities use water taps  1 litre of water used per hand wash  1 mL of soap per hand wash  3 ml of hand sanitiser per hand wash. |
| **Vaccine dose** | Assumptions:   - 15% wastage - 10% markup for freight cost (cost of delivering doses to country) |
| **Vaccine delivery** | **Ethiopia**   - 14 days of vaccine delivery annually, split into two 7-day campaigns - Doses are delivered in facilities by two nurses, one health extension worker, one data collector, and one support staff per site per day - One syringe, one alcohol swab, plaster, and dry swab per dose - 200 doses delivered per facility per day (1 minute spent handwashing, 5 minutes per dose) - Two tables in facility-based delivery, six chairs for staff members plus 10 chairs for the waiting area and for individuals receiving vaccines |
|  | **Nigeria**   - Doses are delivered in facilities by three nurses, two record keepers per site per day - One syringe, one alcohol swab, plaster, and dry swab per dose - 1 minute spent handwashing, 5 minutes per dose - Two tables in facility-based delivery, five chairs for staff members plus 10 chairs for the waiting area and for individuals receiving vaccines |
|  | **South Africa**   - Doses are delivered in facilities by two nurses per site per day - One syringe, one alcohol swab, plaster, and dry swab per dose - 100 doses delivered per facility per day (1 minute spent handwashing, 5 minutes per dose) - Two tables in facility-based delivery, chairs for each staff member plus 10 for the waiting area and for individuals receiving vaccines |
| **Vaccination certificates** | - One certificate per vaccinated individual, assuming 3 minutes for record-keeping per dose. - One FTE staff member per local area level entering data with a laptop into the national vaccine database and office space |
| **Waste management** | - 5L safety box/sharps container: can contain 100 0.5ml syringes (20 syringes per nominal litre). - One biohazardous bag per delivery site per day for used PPE |
| **Pharmacovigilance** | Assuming all staff are working with laptops, and with office space per zone and per region. Assuming a rate of 12.98 serious adverse events per 100,000 doses, each serious AEFI would require 30 minutes of nurse time for management. We assume 15 chairs per vaccination site for vaccinated individuals to wait for 15 mins following immunization.  **Ethiopia**   - One record keeper for data entry, monitoring, and evaluation per zone (73 in total) - One data manager per region (12 in total) overseeing zone record keepers. - One AEFI (adverse event following immunization) kit per month - One AEFI reporting form and one case investigation form per AEFI |
|  | **Nigeria**   - One record keeper for data entry, monitoring, and evaluation per state (37 in total) - One data manager overseeing 37 record keepers. - One AEFI kit per month |
|  | **South Africa**  One data collector for data entry, monitoring and evaluation per local municipality (205 in total)  One data manager per province overseeing local data collectors  One AEFI kit per month  One AEFI reporting form and one case investigation form per AEFI |

### Methods S8. Extrapolating unit costs from base countries to other countries in Africa

To calculate the costs for other African countries, we extrapolated our unit cost per dose estimates for Ethiopia, Nigeria and South Africa to LICs, lower-MICs and upper-MICs, respectively, based on country-specific resource use and health systems data and standard approaches to adjusting prices. Each cost input in the ingredients costing was classified as a tradeable good, non-tradeable good or staff cost.[(46)](https://sciwheel.com/work/citation?ids=9009534&pre=&suf=&sa=0)

Tradeable goods are generally defined as those that can easily be traded in the international market and include goods such as medical or other supplies and medications. To convert costs of tradeable goods from the base country (eg, South Africa) to a ‘second’ country (eg, Namibia), we first converted the prices from local currency to 2021 US$ and then apportioned the percentage of the unit cost that was composed of tradeable goods in 2021 US$ from the base country to the second country.

Non-tradeable goods cannot be easily traded in international markets and generally need to be consumed in the country where they have been produced (eg, buildings and utilities). To convert these, we multiplied the proportion of the unit cost that was defined as non-tradeable (in 2021 US$) by the ratio between the 2021 GDP per capita (adjusted for purchasing power parity, or 'PPP') of the second country and the 2021 GDP per capita (adjusted for PPP) of the base country. Data on GDP per capita (adjusted for PPP) were found in the World Bank database.[(47)](https://sciwheel.com/work/citation?ids=12814611&pre=&suf=&sa=0)

To convert staff costs from a base country to a second country, we used conversion rates from a regression analysis on wages of health workers for 193 countries to predict wages by country income category relative to GDP per capita.[(48)](https://sciwheel.com/work/citation?ids=4941986&pre=&suf=&sa=0) Country-specific wages for physicians, nurses and other health workers were estimated using the conversion rates and respective GDP per capita. Staff costs were extrapolated by multiplying the staff proportion of the unit cost by the ratio between the estimated wages and actual salary levels from the base countries.

Unit costs used for other countries were obtained through extrapolation of these three countries based on country income groups (i.e. LIC, LMIC or UMIC) and cost types (i.e. costs on tradable goods, non-tradable goods, or staff). Tradeable costs were assumed to be constant across countries (e.g. vaccine prices), with non-tradable costs adjusted by country income level. This approach assumes that resource use would be the same within-country income categories (adjusted by population and size) but prices of inputs would vary by country. [(46)](https://sciwheel.com/work/citation?ids=9009534&pre=&suf=&sa=0)

We further adjusted the unit cost per dose to account for the feasible scale of the vaccine roll-out and delivery in each country. Seven activities were adjusted according to scale per country including planning and coordination, technical assistance, training, social mobilisation, vaccine transport, cold chain and pharmacovigilance.

Each cost input for the base countries was assigned a sub-activity level according to where COVID-19 vaccine-specific resources are deployed - first level (eg, national), second level (eg, province), third level (eg, district) and facility level. The type and number of administrative divisions for countries in Africa were gathered from a variety of government and non-government sources, and the number of public health facilities per country was obtained from a comprehensive spatial inventory of public health facilities for 50 countries in Sub-Saharan Africa. [(49)](https://sciwheel.com/work/citation?ids=7237641&pre=&suf=&sa=0)

The type of administrative division in the other countries was matched to their respective base country’s sub-activity level and the unit cost per dose was apportioned accordingly. The unit cost at the sub-activity level was multiplied by an adjustment factor capturing the difference in the number of administrative divisions and health facilities between each country and the base country.

### Methods S9. Extrapolating vaccine unit costs for different roll-out scenarios

From the cross-country vaccine unit costs cross-extrapolation step, we obtained country-level vaccine unit costs by three different roll-out rate levels (275, 826, 2066 doses/ million population-day), two vaccine types (viral vector and mRNA vaccines), and various different program duration length (1 year, 1.5 years, 2 years, 2.5 years). With this raw data we derived a linear model with country-specific coefficients:

(vaccine unit cost) = a + b1*ISO3C + b2*VaccineType +

b3*ProgrammeDuration + b4*RolloutRate

ISO3c and VaccineType are categorical variables; ProgrammeDuration and Rollout Rate are continuous variables. We found that all variable returns statistical significance. This model has a multiple R-squared of 0.9324 and an adjusted R-squared of 0.9277. We used this model to extrapolate vaccine unit costs for additional roll-out scenarios.

Moreover, based on the results from the regression model, we learned that mRNA is positively associated with vaccine unit cost. Both ProgrammeDuration and RolloutRate are negatively associated with the vaccine unit cost due to large initial fixed costs and smaller variable costs.

### Methods S10. ICERs and Proportions of DALYs averted by those above 60 years

There are two possible relationships between ICER_fast and ICER_medium: (a) ICER_fast > ICER_medium; and (b) ICER_fast < ICER_medium. We omitted when ICER_fast == ICER_medium as we want to focus on comparing the extremes. Outcome (b) is intuitively expected, while (a) would be surprising. Outcome (a) essentially implies that medium roll-out rates perform more desirably compared to fast roll-out rates.

One potential hypothesis that can explain this is the contrast in marginal effectiveness between vaccinating older adults versus adults. Older adults (above 60) are more vulnerable to SARS-CoV-2 and are more at risk of progressing to more severe outcomes. Thus, protecting them with vaccines may be associated with higher marginal effectiveness. To test this hypothesis, we relate how ICERs compare by roll-out rates to proportions of DALYs averted by those above 60 years of age (paDALYs). If this hypothesis is valid, we’d expect Outcome (a) to be associated with higher proportions of DALYs averted under medium, compared to fast roll-out rates.

| mRNA vaccines | ICER_fast < ICER_medium  Outcome (2) | ICER_fast > ICER_medium  Outcome (1) |
| --- | --- | --- |
| paDALY_fast < paDALY_medium | 14 | 224 |
| paDALY_fast > paDALY_medium | 6 | 34 |

Fisher’s exact test results in p-value of 0.05.

| Viral vector vaccines | ICER_fast < ICER_medium  Outcome (2) | ICER_fast > ICER_medium  Outcome (1) |
| --- | --- | --- |
| paDALY_fast < paDALY_medium | 67 | 158 |
| paDALY_fast > paDALY_medium | 17 | 19 |

Chi-squared test results in p-value of 0.04.

The higher ICERs under medium roll-out rates compared to fast are associated having higher proportions of DALYs averted by those above 60 years of age. The marginal effectiveness hypothesis is thus validated.

## References

[1.    Liu Y, Pearson CAB, Sandmann FG, Barnard RC, Kim JH, CMMID COVID-19 Working Group, et al. Dosing interval strategies for two-dose COVID-19 vaccination in 13 middle-income countries of Europe: Health impact modelling and benefit-risk analysis. Lancet Reg Health Eur. 2022;17:100381.](https://sciwheel.com/work/bibliography/12801904)

[2.    Mathieu E, Ritchie H, Rodés-Guirao L, Appel C, Giattino C, Hasell J, et al. Coronavirus Pandemic (COVID-19). Our World in Data. 2020;](https://sciwheel.com/work/bibliography/11703906)

[3.    Davies NG, Klepac P, Liu Y, Prem K, Jit M, CMMID COVID-19 working group, et al. Age-dependent effects in the transmission and control of COVID-19 epidemics. Nat Med. 2020;26(8):1205–11.](https://sciwheel.com/work/bibliography/9102939)

[4.    Levin AT, Hanage WP, Owusu-Boaitey N, Cochran KB, Walsh SP, Meyerowitz-Katz G. Assessing the age specificity of infection fatality rates for COVID-19: systematic review, meta-analysis, and public policy implications. Eur J Epidemiol. 2020;35(12):1123–38.](https://sciwheel.com/work/bibliography/10252041)

[5.    Salje H, Tran Kiem C, Lefrancq N, Courtejoie N, Bosetti P, Paireau J, et al. Estimating the burden of SARS-CoV-2 in France. Science. 2020;369(6500):208–11.](https://sciwheel.com/work/bibliography/8898230)

[6.    Davies NG, Barnard RC, Jarvis CI, Russell TW, Semple MG, Jit M, et al. Association of tiered restrictions and a second lockdown with COVID-19 deaths and hospital admissions in England: a modelling study. Lancet Infect Dis. 2021;21(4):482–92.](https://sciwheel.com/work/bibliography/10245997)

[7.    Prem K, Zandvoort KV, Klepac P, Eggo RM, Davies NG, Centre for the Mathematical Modelling of Infectious Diseases COVID-19 Working Group, et al. Projecting contact matrices in 177 geographical regions: An update and comparison with empirical data for the COVID-19 era. PLoS Comput Biol. 2021;17(7):e1009098.](https://sciwheel.com/work/bibliography/11703857)

[8.    United Nations Department of Economic and Social Affairs, Population Division. 2019 World Population Prospects. United Nations: https://population.un.org/wpp; 2019.](https://sciwheel.com/work/bibliography/8436701)

[9.    Pearson CAB, Bozzani F, Procter SR, Davies NG, Huda M, Jensen HT, et al. COVID-19 vaccination in Sindh Province, Pakistan: A modelling study of health impact and cost-effectiveness. PLoS Med. 2021;18(10):e1003815.](https://sciwheel.com/work/bibliography/12468223)

[10.   Davies NG, Kucharski AJ, Eggo RM, Gimma A, Edmunds WJ, Centre for the Mathematical Modelling of Infectious Diseases COVID-19 working group. Effects of non-pharmaceutical interventions on COVID-19 cases, deaths, and demand for hospital services in the UK: a modelling study. Lancet Public Health. 2020;5(7):e375–85.](https://sciwheel.com/work/bibliography/9011259)

[11.   Bi Q, Wu Y, Mei S, Ye C, Zou X, Zhang Z, et al. Epidemiology and transmission of COVID-19 in 391 cases and 1286 of their close contacts in Shenzhen, China: a retrospective cohort study. Lancet Infect Dis. 2020;20(8):911–9.](https://sciwheel.com/work/bibliography/8792615)

[12.   Liu Y, CMMID COVID-19 working group, Funk S, Flasche S. The contribution of pre-symptomatic infection to the transmission dynamics of COVID-2019 [version; peer review: 3 approved]. Wellcome Open Res. 2020;](https://sciwheel.com/work/bibliography/14346765)

[13.   Linton NM, Kobayashi T, Yang Y, Hayashi K, Akhmetzhanov AR, Jung SM, et al. Incubation Period and Other Epidemiological Characteristics of 2019 Novel Coronavirus Infections with Right Truncation: A Statistical Analysis of Publicly Available Case Data. J Clin Med. 2020;9(2).](https://sciwheel.com/work/bibliography/8415907)

[14.   Nishiura H, Linton NM, Akhmetzhanov AR. Serial interval of novel coronavirus (COVID-19) infections. Int J Infect Dis. 2020;93:284–6.](https://sciwheel.com/work/bibliography/8415585)

[15.   Hall VJ, Foulkes S, Charlett A, Atti A, Monk EJM, Simmons R, et al. SARS-CoV-2 infection rates of antibody-positive compared with antibody-negative health-care workers in England: a large, multicentre, prospective cohort study (SIREN). Lancet. 2021;397(10283):1459–69.](https://sciwheel.com/work/bibliography/14341998)

[16.   Torres-Rueda S, Sweeney S, Bozzani F, Naylor NR, Baker T, Pearson C, et al. Stark choices: exploring health sector costs of policy responses to COVID-19 in low-income and middle-income countries. BMJ Glob Health. 2021;6(12).](https://sciwheel.com/work/bibliography/12767344)

[17.   Google Inc. COVID-19 Community Mobility Reports (2020-2022). Google Inc.: https://www.google.com/covid19/mobility/; 2022.](https://sciwheel.com/work/bibliography/11703648)

[18.   Hale T, Angrist N, Goldszmidt R, Kira B, Petherick A, Phillips T, et al. A global panel database of pandemic policies (Oxford COVID-19 Government Response Tracker). Nat Hum Behav. 2021;5(4):529–38.](https://sciwheel.com/work/bibliography/10678231)

[19.   World Bank. GDP per capita (current US$) | Data. World Bank: https://data.worldbank.org/indicator/NY.GDP.PCAP.CD; 2022.](https://sciwheel.com/work/bibliography/13581342)

[20.   World Bank. Domestic general government health expenditure per capita (current US$) | Data. World Bank: https://data.worldbank.org/indicator/SH.XPD.GHED.PC.CD; 2022.](https://sciwheel.com/work/bibliography/12819804)

[21.   World Bank Data Help Desk. World Bank Country and Lending Groups. World Bank: https://datahelpdesk.worldbank.org/knowledgebase/articles/906519-world-bank-country-and-lending-groups; 2022.](https://sciwheel.com/work/bibliography/13585585)

[22.   Liu Y, Sandmann FG, Barnard RC, Pearson CAB, Pastore R, Pebody R, et al. Optimising health and economic impacts of COVID-19 vaccine prioritisation strategies in the WHO European Region: a mathematical modelling study. Lancet Reg Health Eur. 2022;12:100267.](https://sciwheel.com/work/bibliography/12467362)

[23.   World Health Organization. The Pfizer BioNTech (BNT162b2) COVID-19 vaccine: What you need to know. World Health Organization. 2022;](https://sciwheel.com/work/bibliography/12766627)

[24.   UK Health Security Agency. 14a. Guidance COVID-19: the Green Book. UK Health Security Agency; 2022.](https://sciwheel.com/work/bibliography/12766626)

[25.   World Health Organization. WHO SAGE Roadmap for prioritizing uses of COVID-19 vaccines: An approach to optimize the global impact of COVID-19 vaccines, based on public health goals, global and national equity, and vaccine access and coverage scenarios. World Health Organization; 2022.](https://sciwheel.com/work/bibliography/13556792)

[26.   Gooding K, Webster J, Wiafe N, Kumar V. Real-time assessment of UNICEF’s ongoing response to COVID-19 in eastern and southern Africa. Oxford Policy Management; 2021.](https://sciwheel.com/work/bibliography/13556794)

[27.   World Health Organization. Achieving 70% COVID-19 Immunization Coverage by Mid-2022. World Health Organization. 2021;](https://sciwheel.com/work/bibliography/12801839)

[28.   Robinson E, Jones A, Lesser I, Daly M. International estimates of intended uptake and refusal of COVID-19 vaccines: A rapid systematic review and meta-analysis of large nationally representative samples. Vaccine. 2021;39(15):2024–34.](https://sciwheel.com/work/bibliography/11230457)

[29.   Barnard RC, Davies NG, Centre for Mathematical Modelling of Infectious Diseases COVID-19 working group, Jit M, Edmunds WJ. Modelling the medium-term dynamics of SARS-CoV-2 transmission in England in the Omicron era. Nat Commun. 2022;13(1):4879.](https://sciwheel.com/work/bibliography/13511412)

[30.   Pearson CAB, Silal SP, Li MWZ, Dushoff J, Bolker BM, Abbott S, et al. Bounding the levels of transmissibility & immune evasion of the Omicron variant in South Africa. medRxiv. 2021;](https://sciwheel.com/work/bibliography/12346822)

[31.   Grint DJ, Wing K, Houlihan C, Gibbs HP, Evans SJW, Williamson E, et al. Severity of Severe Acute Respiratory System Coronavirus 2 (SARS-CoV-2) Alpha Variant (B.1.1.7) in England. Clin Infect Dis. 2022;75(1):e1120–7.](https://sciwheel.com/work/bibliography/12936474)

[32.   Public Health England. SARS-CoV-2 variants of concern and variants under investigation in England - Technical Briefing 14. Public Health England; 2021.](https://sciwheel.com/work/bibliography/12936483)

[33.   UK Health Security Agency. SARS-CoV-2 variants of concern and variants under investigation in England - Technical briefing: Update on hospitalisation and vaccine effectiveness for Omicron VOC-21NOV-01 (B.1.1.529) . UK Health Security Agency; 2021.](https://sciwheel.com/work/bibliography/12936429)

[34.   Iuliano AD, Brunkard JM, Boehmer TK, Peterson E, Adjei S, Binder AM, et al. Trends in Disease Severity and Health Care Utilization During the Early Omicron Variant Period Compared with Previous SARS-CoV-2 High Transmission Periods - United States, December 2020-January 2022. MMWR Morb Mortal Wkly Rep. 2022;71(4):146–52.](https://sciwheel.com/work/bibliography/12350278)

[35.   Pouwels KB, Pritchard E, Matthews PC, Stoesser N, Eyre DW, Vihta K-D, et al. Effect of Delta variant on viral burden and vaccine effectiveness against new SARS-CoV-2 infections in the UK. Nat Med. 2021;27(12):2127–35.](https://sciwheel.com/work/bibliography/11880224)

[36.   UK Health Security Agency. COVID-19 vaccine surveillance report Week 2. UK Health Security Agency; 2022.](https://sciwheel.com/work/bibliography/12936505)

[37.   Krammer F, Srivastava K, Alshammary H, Amoako AA, Awawda MH, Beach KF, et al. Antibody Responses in Seropositive Persons after a Single Dose of SARS-CoV-2 mRNA Vaccine. N Engl J Med. 2021;384(14):1372–4.](https://sciwheel.com/work/bibliography/10652101)

[38.   Mullen K, Ardia D, Gil D, Windover D, Cline J. deoptim : an *r* package for global optimization by differential evolution. J Stat Softw. 2011;40(6).](https://sciwheel.com/work/bibliography/12444549)

[39.   Leclerc QJ, Fuller NM, Keogh RH, Diaz-Ordaz K, Sekula R, Semple MG, et al. Importance of patient bed pathways and length of stay differences in predicting COVID-19 hospital bed occupancy in England. BMC Health Serv Res. 2021;21(1):566.](https://sciwheel.com/work/bibliography/12767052)

[40.   Wyper GMA, Assunção RMA, Colzani E, Grant I, Haagsma JA, Lagerweij G, et al. Burden of Disease Methods: A Guide to Calculate COVID-19 Disability-Adjusted Life Years. Int J Public Health. 2021;66:619011.](https://sciwheel.com/work/bibliography/12433748)

[41.   Salomon JA, Haagsma JA, Davis A, de Noordhout CM, Polinder S, Havelaar AH, et al. Disability weights for the Global Burden of Disease 2013 study. Lancet Glob Health. 2015;3(11):e712-23.](https://sciwheel.com/work/bibliography/854002)

[42.   Haagsma JA, Maertens de Noordhout C, Polinder S, Vos T, Havelaar AH, Cassini A, et al. Assessing disability weights based on the responses of 30,660 people from four European countries. Popul Health Metr. 2015;13:10.](https://sciwheel.com/work/bibliography/2171766)

[43.   Griffiths U, Adjagba A, Attaran M, Hutubessy R, Van de Maele N, Yeung K, et al. Costs of delivering COVID-19 vaccine in 92 AMC countries Updated estimates from COVAX Working Group on delivery costs. World Health Organization; 2021.](https://sciwheel.com/work/bibliography/12814598)

[44.   Global Health Centre. COVID-19 Vaccine Access. Geneva Graduate Institute; 2022.](https://sciwheel.com/work/bibliography/14347149)

[45.   Vassall A, Sweeney S, Kahn J, Gomez Guillen G, Bollinger L, Marseille E, et al. Reference case for estimating the costs of global health services and interventions. Global Health Cost Consortium; 2017.](https://sciwheel.com/work/bibliography/12814602)

[46.   Turner HC, Lauer JA, Tran BX, Teerawattananon Y, Jit M. Adjusting for inflation and currency changes within health economic studies. Value Health. 2019;22(9):1026–32.](https://sciwheel.com/work/bibliography/9009534)

[47.   World Bank. World Bank Open Data. World Bank: https://data.worldbank.org/; 2022.](https://sciwheel.com/work/bibliography/12814611)

[48.   Serje J, Bertram MY, Brindley C, Lauer JA. Global health worker salary estimates: an econometric analysis of global earnings data. Cost Eff Resour Alloc. 2018;16:10.](https://sciwheel.com/work/bibliography/4941986)

[49.   Maina J, Ouma PO, Macharia PM, Alegana VA, Mitto B, Fall IS, et al. A spatial database of health facilities managed by the public health sector in sub Saharan Africa. Sci Data. 2019;6(1):134.](https://sciwheel.com/work/bibliography/7237641)
